# UV Laser-Induced Photodecomposition of Matrix-Isolated Salicylhydroxamic Acid: Identification of New Isocyanate Complexes

**DOI:** 10.3390/molecules29040862

**Published:** 2024-02-15

**Authors:** Magdalena Sałdyka, Zofia Mielke

**Affiliations:** Faculty of Chemistry, University of Wroclaw, F. Joliot-Curie 14, 50-383 Wrocław, Poland

**Keywords:** salicylhydroxamic acid, photolysis, matrix isolation, DFT, isocyanate, molecular complex

## Abstract

Photochemical reactions of salicylhydroxamic acid were induced using tunable UV laser radiation followed by FTIR spectroscopy. Four pairs of co-products were experimentally found to appear in the photolysis: C_6_H_4_(OH)NCO⋯H_2_O (1), C_6_H_4_(OH)C(O)N⋯H_2_O (2), C_6_H_4_(OH)_2_⋯HNCO (3), and C_6_H_4_(OH)NHOH⋯CO (4). The comparison of the theoretical spectra with the experimental ones allowed us to determine the structures of the complexes formed in the matrices. The mechanisms of the reaction channels leading to the formation of the photoproducts were proposed. It was concluded that the first step in the formation of the complexes (1), (2), and (3) was the scission of the N-O bond, whereas the creation of complex (4) was due to cleavage of the C-N bond.

## 1. Introduction

Since their discovery, hydroxamic acids have been the subject of still-growing interest due to their rich chemistry and implication in a wide spectrum of biological activities. A number of hydroxamic acids act as antibiotics, antitumor and antifungal agents, and specific enzyme inhibitors. Due to their ability to form stable chelates with a variety of metal ions, hydroxamic acids play a key role as bioligands in the microbial transport of iron. Also, their possible NO-releasing properties make up their versatility in biological activities [1,2,3]. Salicylhydroxamic acid (SHA) is an inhibitor of urease and peroxidase as well as a strong inhibitor of cyclooxygenase [4]. It also acts as an efficient ligand in mixed ternary complexes with ribonucleotides and metal (II) ions [5,6]. In addition, the antibacterial properties of SHA and its halogenated derivatives, which showed anti-tuberculosis activity in in vivo and in vitro tests, have been investigated [7,8]. SHA and its metal complexes have also shown antifungal or antiparasitic activity [9].

**Figure 1:**
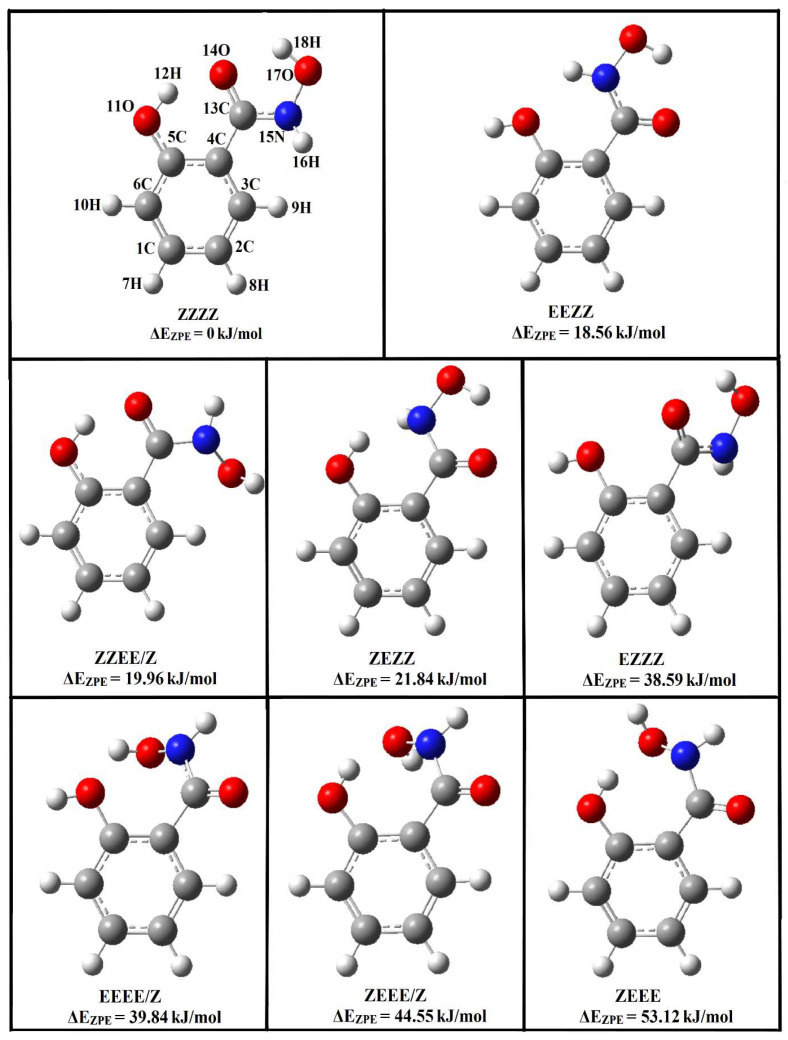
Structures of Z and E isomers of the keto forms of salicylhydroxamic acid optimized at the B3LYPD3/6-311++G(2d,2p) level; the relative zero-point vibrational corrected energies are given.

A salicylhydroxamic acid structure may occur in the form of keto tautomers, 1Z and 1E, and enol tautomers, 2Z and 2E [9,10,11,12,13,14]. X-ray crystallography indicates that the 1Z-keto tautomer is the stable crystal structure for SHA, with an intramolecular hydrogen bond between the oxygen atom coming from the hydroxyl group attached directly to the ring and the hydrogen atom attached to the nitrogen from the hydroxamic group. The conformation of the hydroxamic group is synperiplanar, and the torsional angle O-C-N-O is 6.3°. Stabilization of molecular packing occurs through the formation of hydrogen bonds and van der Waals interactions [15]. Recently, spectroscopic and theoretical investigations have been performed for SHA. NMR studies indicate that, in acetone, only the 1E form of SHA exists, while B3LYP/aug-cc-pVDZ calculations evidence that, in the gas phase, the 1Z form is predominant [16]. Other ^1^H, ^13^C NMR, and COSY NMR studies show that SHA adopts the Z-keto conformation due to its strong interaction with solvent molecules [17]. Infrared matrix isolation studies have confirmed the results of calculations and have shown that SHA exists in the 1Z-keto form in the gas phase and in solid argon (Z,Z,Z,Z is the full description of the tautomer, taking into account the rotation around the C-O, C-C, C-N, and N-O bonds; see Figure 1) [18].

Previous research on the photochemistry of hydroxamic acids indicates that the main photolysis path of aromatic hydroxamic acids starts with the scission of the N-O bond, with the formation of amides and anilides [19]. However, irradiation of some naphthalenehydroxamic acids [19] produces acylaminoxyl radicals, which are not the result of breaking the N-O bond but of other rearrangements within the molecule. Acylaminoxyl radical formation is the first step of the photodissociation of *N*-substituted hydroxamic acids with the hydroxyl group [20]. Studies on the photolysis of hydroxamic acids isolated in low-temperature matrices carried out in our group [21,22,23] showed that UV-Vis irradiation of formohydroxamic and acetohydroxamic acids or N-hydroxyurea leads to the cleavage of the N-O or C-N bonds in these molecules. The photolysis of salicylhydroxamic acid in solid argon has been reported [18]. Broad-band UV irradiation of a mercury lamp has been used to obtain the photodegradation reactions of SHA, and it turned out that *o*-hydroxyphenylisocyanate is the main photolysis product in an argon matrix. In addition, an intermediate compound, whose structure lies between nitrene and oxazirene, has also been identified. As noted above, solution photolysis of hydroxamic acids generally leads to the corresponding amides or anilides [19]. Room-temperature photolysis at 300 nm of SHA in solutions (30% methanol–water) was performed in ref. [18]. The electrospray mass spectrometry used to identify the photolysis products showed a decrease in the SHA molecular ion signal (*m*/*z* = 154.8 ± 1.0) after 30 min and its disappearance after 60 min of irradiation. At the same time, a peak at *m*/*z* = 138.9 ± 1.0 was observed, which started to grow and became dominant in the spectrum after 30 min. Since the photodegradation of hydroxamic acids in a solution generates amides (salicylamide has a mass of 137.1), it was concluded that only salicylamide is the photoproduct of the irradiation of SHA.

The aim of this work was to investigate the UV laser-induced phototransformation of salicylhydroxamic acid in argon and nitrogen matrices in order to check whether tuned irradiation provides additional information on the photochemical behavior of the SHA/Ar system in relation to the broad-band UV experiment reported earlier. The performed study demonstrated that the main SHA photoproducts identified in the previous work—C_6_H_4_(OH)NCO and C_6_H_4_(OH)C(O)N—form a number of interesting complexes with H_2_O; moreover, C_6_H_4_(OH)_2_ and HNCO and C_6_H_4_(OH)NHOH and CO are also formed as co-products during SHA photolysis. The obtained spectroscopic data and their interpretation, supported by B3LYPD3/6–311++G(2d,2p) calculations, are presented in this paper.

Low-temperature matrix isolation coupled with infrared spectroscopy has been efficiently used to study conformational changes or molecular interactions [24,25]. IR spectroscopy provides a lot of valuable information about the structural properties of a studied compound. Usually, a gas-phase equilibrium conformational (or tautomeric) population is frozen in a solid noble gas cryogenic matrix, with the concentration of the matrix material low enough to assure that the molecules are well isolated. The matrix IR spectra are quite well modeled by quantum–chemical calculations in the gas phase due to the low bandwidth of the species isolated in the matrices and due to the inhibited rotation and very weak interactions between the studied molecules and the matrix environment. For this reason, quantum–chemical calculations of vibration frequencies and intensities are performed for the studied systems to support experimental data interpretation. This makes isolation in low-temperature matrices together with theoretical calculations an ideal tool for determining the molecular structure of molecules/complexes.

## 2. Results and Discussion

### 2.1. Identification of Photoproducts

The OPO tunable laser was used for the UV irradiation of SHA isolated in argon and nitrogen matrices. The irradiations started at a wavelength of 400 nm, and the wavelength was gradually decreased. The infrared spectrum of the matrix was measured after each exposure. SHA photolysis started at 370 nm but turned out to be very slow. The best results were obtained for exposure at a wavelength of 340 nm. A clear decrease in intensity of the SHA bands, the appearance of new absorptions, and an increase in their intensities were observed. Figure S1 presents the behavior of the main photoproduct bands during irradiation. 

**Figure 2:**
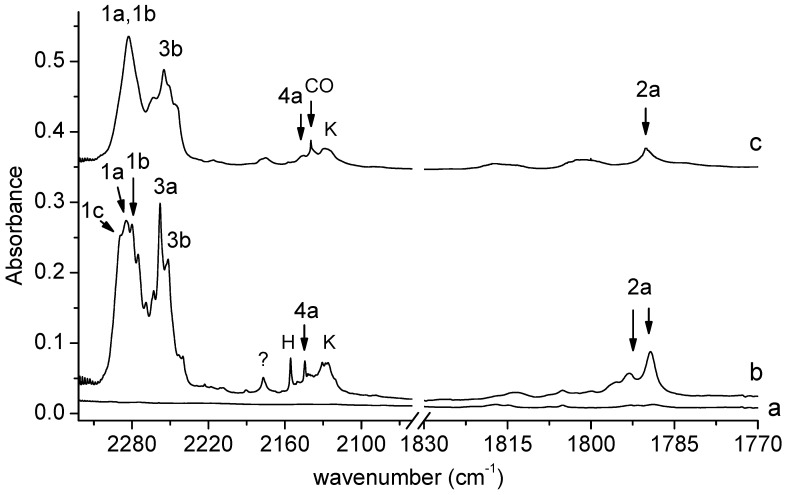
The 2322–1770 cm^−1^ region of the spectra of SHA/Ar matrix after deposition (a) and after 150 min irradiation of the deposited matrix at λ = 340 nm (b). The spectra of SHA/N_2_ matrix after 150 min irradiation at λ = 340 nm (c). The bands due to the complexes are labeled by numbers; the label CO indicates bands due to carbon monoxide; H—bands due to the HNCO-CO complex; K—bands due to the ketoketene–NH_2_OH complex.

Figure 2 shows the 2322–1770 cm^−1^ spectral region of SHA isolated in solid argon/nitrogen recorded directly after matrix deposition and after matrix exposure to λ = 340 nm radiation. This is the key region as far as the identification of photolysis products is concerned. We can distinguish four groups of bands that grow in this region after the SHA/Ar(N_2_) matrix is exposed to λ = 340 nm radiation emitted by the OPO. Two groups involve broad absorptions extending in the 2300–2270 and 2270–2230 cm^−1^ regions, with subpeaks at 2289.0, 2284.0, 2280.0, 2275.0, 2258.0, and 2252.0 cm^−1^ in solid argon and at 2283.0 and 2255.0 cm^−1^ in solid nitrogen. Another group of bands occurs in the region of the CO molecule vibration. The main photoproduct band appears at 2144.0 cm^−1^ in Ar or at 2146.0 cm^−1^ in N_2_. The other group involves a doublet at 1793.0 and 1789.0 cm^−1^ in the argon matrix, and a band at 1790.0 cm^−1^ in the nitrogen matrix. The four groups of bands corresponding to the four pairs of photoproducts will be discussed below.

**Figure 3:**
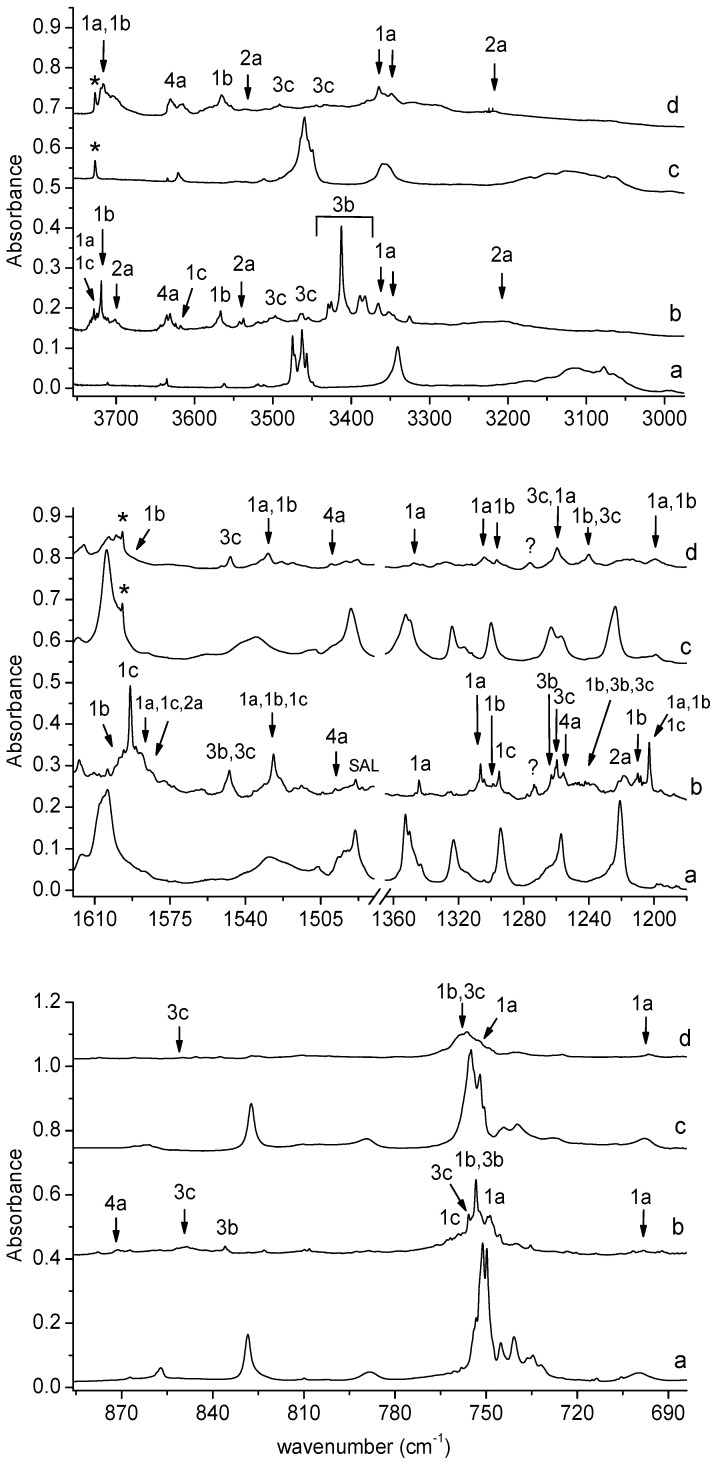
The 3750–2980, 1620–1180, and 885–685 cm^−1^ regions of the spectra of SHA/Ar matrix after deposition (a) and after 150 min irradiation of the deposited matrix at λ = 340 nm (b). The spectra of SHA/N_2_ matrix after deposition (c) and after 150 min irradiation at λ = 340 nm (d). The labels * indicate bands due to water contamination; SAL—band due to salicylamide.

The broad absorption appearing after irradiation in the 2300–2270 cm^−1^ region (bands of group 1) indicates the formation of an isocyanate derivative during SHA photolysis. The observed subpeaks (2289.0, 2284.0, 2280.0, and 2275.0 cm^−1^ in solid argon and at 2283.0 cm^−1^ in solid nitrogen) are assigned to the C_6_H_4_(OH)NCO complex with H_2_O, whose presence is also manifested in the OH stretching and OH bending regions of the spectra recorded after SHA irradiation (see Figure 3).

The weak bands at 1793.0 and 1789.0 cm^−1^ in an argon matrix and at 1790.0 cm^−1^ in a nitrogen matrix (bands of group 2) indicate the formation of an intermediate compound between nitrene and oxazirene structure (C_6_H_4_(OH)C(O)N) in a complex with H_2_O. Its formation was reported previously in a paper on the photodegradation of SHA triggered by broad UV irradiation [18]. It can be noticed that only two of the four main products of the photolysis of SHA identified here were described in [18]: C_6_H_4_(OH)NCO and C_6_H_4_(OH)C(O)N; the presence of water as the co-product of photolysis and the complexation of the above molecules with water were not reported.

Two strong bands at 2258.0 and 2252.0 cm^−1^ in solid Ar or at 2255 cm^−1^ in solid N_2_ (bands of group 3) observed in the region of isocyanic acid vibration [26,27] are attributed to the perturbed HNCO molecule in a complex with C_6_H_4_(OH)_2_; the bands corresponding to the 1,2-dihydroxybenzene molecule in this complex can be seen in Figure 3.

The appearance of new bands (Figure 2) in the vicinity of the carbon monoxide molecule band at 2138.5 in Ar or at 2139.8 cm^−1^ in N_2_ [28,29] (bands of group 4) indicates a similar photolysis mechanism (i.e., scission of the N-O bond) to that observed for formohydroxamic or acetohydroxamic acids. For both acids, hydroxylamine (NH_2_OH) or *N*-methylhydroxylamine (CH_3_NHOH) occurred as photoproducts, respectively [21,22], together with the CO molecule. So, the detachment of the CO molecule from SHA would create N-*o*-hydroxyphenylhydroxylamine (C_6_H_4_(OH)NHOH) as the co-product, and two co-products trapped in one matrix cage interact with each other to form a complex. Therefore, the new band identified in the range of the CO molecule vibration can be attributed to the perturbed band of CO in a complex with C_6_H_4_(OH)NHOH.

In addition to the absorptions attributed to groups 1, 2, 3, and 4, additional weaker bands appeared in the spectra of the photolyzed matrices. They were assigned on the basis of the literature data to the salicylaldehyde, salicylamide, and ketoketene molecules and to the H_2_O-CO and HNCO-CO complexes (see Section 2.3. Other photolysis products section). In the following paragraphs, a comparison of the calculated and experimental spectra for the C_6_H_4_(OH)NCO-H_2_O, C_6_H_4_(OH)C(O)N-H_2_O, C_6_H_4_(OH)_2_-HNCO, and C_6_H_4_(OH)NHOH-CO complexes will be presented, which provides evidence of the formation of these complexes in the matrices. 

#### 2.1.1. Formation of the C_6_H_4_(OH)NCO-H_2_O Complex

**Figure 4:**
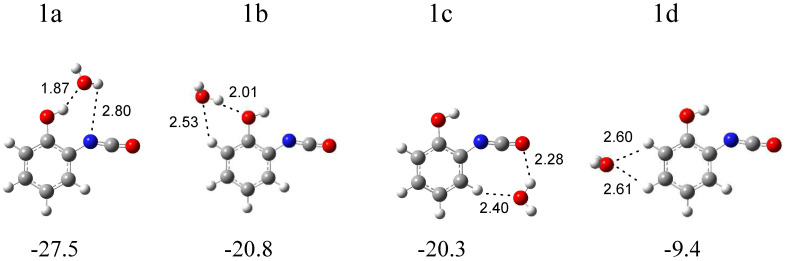
B3LYPD3/6-311++G(2d,2p) optimized structures of the C_6_H_4_(OH)NCO-H_2_O complex. The interaction energies ΔE^CP^ (in kJ mol^−1^) and selected bond distances (in Å) are given.

In Figure 4, four local minima calculated for the *o*-hydroxyphenyl isocyanate complex with water are presented. Structure 1a (∆E^CP^ = −27.5 kJ mol^−1^) is characterized by the presence of a hydrogen bond between the OH group of the isocyanate derivative and the oxygen atom of water. In structure 1b (∆E^CP^ = −20.8 kJ mol^−1^), water plays the role of proton donor towards the oxygen atom of the hydroxyl group of C_6_H_4_(OH)NCO and, in the slightly less stable structure 1c (∆E^CP^ = −20.3 kJ mol^−1^), towards the oxygen atom of the NCO group of isocyanate. Additionally, in structures 1a and 1b, the weak H_2_O∙∙∙H-C(phenyl) interaction is present. In the 1d structure (∆E^CP^ = −9.4 kJ mol^−1^), H_2_O interacts with the CH groups of the phenyl ring of C_6_H_4_(OH)NCO. The full sets of harmonic vibrational wavenumbers of the optimized structures are presented in Table S1. A careful inspection of all the calculated data and comparison of the experimental (bands of group 1) and calculated spectra show with certainty that three complexes are formed in the matrices: 1a and 1b, with water playing the role of proton acceptor or proton donor in the OH∙∙∙O(H_2_) or (H)OH∙∙∙OH interactions, respectively, and structure 1c, with water interacting with the oxygen atom of the NCO and CH groups of the isocyanate molecule. In Table 1, the theoretical wavenumber shifts: ∆ν_cal_ = (ν_complex_ − ν_monomer_)_cal_ for the 1a, 1b, and 1c structures are compared with the experimental ones, ∆ν_exp_ = (ν_complex_ − ν_monomer_)_exp_. Due to the lack of an experimental spectrum for the C_6_H_4_(OH)NCO monomer in the literature, the calculated anharmonic wavenumbers were taken for the ∆ν_exp_ calculations. The anharmonic wavenumbers calculated for C_6_H_4_(OH)NCO are presented in Table S2. The experimental wavenumbers for the H_2_O molecule were taken from ref. [30]. The experimental ν_as_NCO vibration of C_6_H_4_(OH)NCO was found in ref. [31].

In the region of the NCO asymmetric stretching vibration (see Figure 2), a strong band with several subpeaks in an argon matrix (at 2289.0, 2284.0, 2280.0, 2275.0 cm^−1^) and one broad absorption in a nitrogen matrix (at 2283.0 cm^−1^) grew after matrix irradiation, which suggests the formation of more than one optimized structure in the irradiated matrices. The position of these bands in solid argon allowed for grouping them and enables us to draw a conclusion about the presence of three structures—1a, 1b and 1c—in the matrix. The 2285.0 cm^−1^ band was assigned to complex 1a; the bands at 2280 and 2275.0 cm^−1^ were assigned to complex 1b; whereas, the absorption at 2289.0 cm^−1^ was attributed to structure 1c. The appearance of one broad band at 2283.0 cm^−1^ in the region of ν_as_NCO vibration in solid nitrogen did not help us to clearly distinguish the three structures; it seems that the close position of the very strong bands originating from the complexes (calculated intensities = 1577, 1684, and 1660 km mol^−1^ for 1a, 1b, and 1c, respectively) resulted in the formation of one very broad absorption in the nitrogen matrix spectrum. The suggestion of the formation of three complexes is strongly supported by the appearance of three absorptions (alternatively with subpeaks) for the νC=C, δCH, νC-O, δOH, and γCH vibrations of C_6_H_4_(OH)NCO and for the ν_3_ and ν_2_ H_2_O vibrations. As one can see in Table 1, the experimental shifts are in accord with the theoretical ones both for *o*-hydroxyphenyl isocyanate and for the water molecules.

#### 2.1.2. Formation of the C_6_H_4_(OH)C(O)N-H_2_O Complex

**Figure 5:**
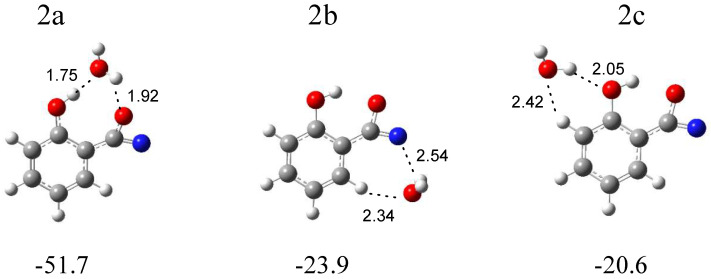
B3LYPD3/6-311++G(2d,2p) optimized structures of the C_6_H_4_(OH)C(O)N-H_2_O complex. The interaction energies ΔE^CP^ (in kJ mol^−1^) and selected bond distances (in Å) are given.

In Figure 5, three local minima optimized for the C_6_H_4_(OH)C(O)N (the intermediate compound between nitrene and the oxazirene structure) complex with water are shown. In the structure 2a (∆E^CP^ = −51.7 kJ mol^−1^), water simultaneously plays the role of proton donor and proton acceptor, and two OH∙∙∙O hydrogen bonds are formed. In the structure 2b (∆E^CP^ = −23.9 kJ mol^−1^), water interacts with the nitrogen atom of the CNO group; in addition, a weak H_2_O∙∙∙H-C(phenyl) interaction is present. In 2c (∆E^CP^ = −20.6 kJ mol^−1^), H_2_O interacts with the hydroxyl group of C_6_H_4_(OH)C(O)N, forming a (H)OH∙∙∙OH hydrogen bond. Based on the comparison of the experimental (bands of group 2) and calculated spectra for the three structures, it can be concluded that the complex structure 2a is trapped in the matrix with the C_6_H_4_(OH)C(O)N molecule acting as a proton donor toward the H_2_O molecule. Table 2 shows the comparison of the theoretical wavenumber shifts for the 2a, 2b, and 2c structures with the experimental ones. The H_2_O monomer wavenumbers were taken from reference [30]. The anharmonic wavenumbers calculated for C_6_H_4_(OH)C(O)N are presented in Table S4. The full sets of the vibrational wavenumbers of the optimized structures are presented in Table S3.

The weak but well-defined bands at 1793.0 and 1789.0 cm^−1^ in an argon matrix and at 1790.0 cm^−1^ in a nitrogen matrix (bands of group 2) indicate the formation of C_6_H_4_(OH)C(O)N in a complex with H_2_O. The wavenumber shift estimation of these bands with respect to the monomer is not sufficient to determine the structure of the complex due to small differences between the calculated wavenumber shift values of ν_as_CCN for 2a, 2b, and 2c. However, the presence of the ν_s_OH (H_2_O) and νOH (C_6_H_4_(OH)C(O)N) bands at 3537 cm^−1^ in Ar and 3535 cm^−1^ in N_2_ and 3208 cm^−1^ in Ar and 3218 cm^−1^ in N_2_, respectively, allows us to determine with high probability the formation of the most stable complex 2a in both matrices.

#### 2.1.3. Formation of the C_6_H_4_(OH)_2_-HNCO Complex

**Figure 6:**
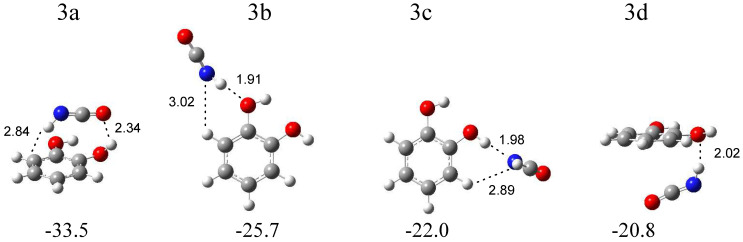
B3LYPD3/6-311++G(2d,2p) optimized structures of the C_6_H_4_(OH)_2_-HNCO complex. The interaction energies ΔE^CP^ (in kJ mol^−1^) and selected bond distances (in Å) are given.

The calculations resulted in four local minima on the potential energy surface of the isocyanic acid–1,2-dihydroxybenzene system that correspond to the stable structures presented in Figure 6. In the most stable structure, 3a (∆E^CP^ = −33.5 kJ mol^−1^), the NH group of HNCO interacts with the benzene ring of 1,2-dihydroxybenzene (NH∙∙∙π type interaction). In turn, in the 3b configuration (∆E^CP^ = −25.7 kJ mol^−1^), the NH group of HNCO serves as a proton donor toward the oxygen atom of 1,2-dihydroxybenzene. The structure 3c (∆E = −22.0 kJ mol^−1^) is stabilized by the OH∙∙∙N hydrogen bond formed between the OH group of C_6_H_4_(OH)_2_ and the nitrogen atom of HNCO. In the structure 3d (∆E = −22.8 kJ mol^−1^), an interaction between the NH group of HNCO and the oxygen atom of C_6_H_4_(OH)_2_ is present, with both molecules positioned almost parallel to each other. The comparison of the experimental spectra (bands assigned to the group 3) with those calculated for the four structures evidences that two complex structures, 3b and 3c, are trapped in the matrix with the C_6_H_4_(OH)_2_ molecule acting as a proton acceptor or a proton donor toward the NH group of HNCO. In Table 3, the theoretical wavenumber shifts for the 3a, 3b, and 3c structures are compared with the experimental ones. The HNCO and C_6_H_4_(OH)_2_ monomer wavenumbers were taken from references [26,27,32]. The anharmonic wavenumbers calculated for C_6_H_4_(OH)_2_ are presented in Table S6. The full sets of the vibrational wavenumbers of the optimized structures are presented in Table S5. In the key spectral region of the irradiated matrices, two bands at 2258.0 and 2252.0 cm^−1^ were identified for the ν_as_NCO stretching vibration of the complexed isocyanic acid; the bands are red-shifted (∆ν_exp_ = −1 and −7 cm^−1^) with respect to the corresponding absorption of the HNCO monomer isolated in an argon matrix [24]. The observed shifts of the two bands suggest that two structures, 3b and 3c, are formed in the argon matrix; the 2258.0 cm^−1^ band corresponds to the 3b structure (∆ν_theor_ = 0 cm^−1^), and the 2252.0 cm^−1^ absorption band is attributed to the 3c configuration (∆ν_theor_ = −2 cm^−1^). In the nitrogen matrix, only one band at 2255.0 cm^−1^ was identified in this spectral region.

The wavenumber shift of the 2255.0 cm^−1^ band in the N_2_ matrix (∆ν_exp_ = −10 cm^−1^) implies that, in this environment, only the 3c complex is formed (∆ν_theor_ = −2 cm^−1^). A similar absorption pattern can be observed in other spectral regions, especially in the high-wavenumber region. As one can see in Figure 3, the presence of strong νNH vibration bands for complex 3b in the argon matrix (at ca. 3405 cm^−1^) and the complete absence of these bands in the nitrogen matrix are the most visible features in the spectrum. Only a very weak band of complex 3c at 3497 cm^−1^ in solid Ar or at 3492 cm^−1^ in solid N_2_ can be identified in both matrices. These observations provide strong evidence for the formation of only one complex structure of C_6_H_4_(OH)_2_-HNCO in a nitrogen matrix and indicates that the matrix can influence the nature of the formed complex [33]. On the other hand, the production of the metastable complex in the matrices (3b or 3c), but not the global minimum structure (3a), by UV irradiation of the precursor molecule has been previously observed, for example, after the photodissociation of simple hydroxamic acids [21,22,23], formaldoxime [34], or acetamide [35].

#### 2.1.4. Formation of the C_6_H_4_(OH)NHOH-CO Complex

**Figure 7:**
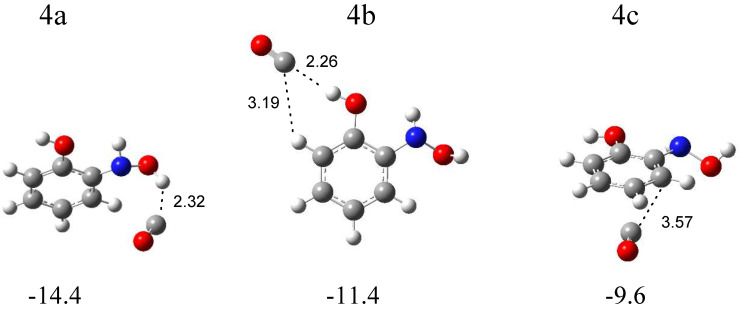
B3LYPD3/6-311++G(2d,2p)-optimized structures of the C_6_H_4_(OH)NHOH-CO complex. The interaction energies ΔE^CP^ (in kJ mol^−1^) and selected bond distances (in Å) are given.

The calculations resulted in three local minima on the potential energy surface of the N-*o*-hydroxyphenylhydroxylamine (SalHy)–carbon monoxide system that correspond to the stable structures presented in Figure 7. In 4a (∆E^CP^ = −14.4 kJ mol^−1^) and 4b (∆E^CP^ = −11.4 kJ mol^−1^) structures, a similar OH∙∙∙CO interaction is present. In the 4a complex, the OH group comes from the NHOH group of SalHy, and, in the 4b structure, the OH group is bonded to the phenyl ring. In the 4c structure (∆E^CP^ = −9.6 kJ mol^−1^), the CO molecule weakly interacts with the aromatic ring. Table S7 collects the full sets of the vibrational wavenumbers of the optimized complexes; Table S8 gathers the anharmonic wavenumbers calculated for SalHy. In Table 4, the theoretical wavenumber shifts for the 4a, 4b, and 4c structures are shown, together with the experimental ones. The experimental wavenumber for the CO molecule was taken from references [28,29].

The comparison of the experimental (bands of group 4) and calculated spectra for the three structures proves that complex 4a is formed in the matrices with the (NH)OH∙∙∙CO hydrogen bond. The appearance of new bands in the range of νOH and δOH vibrations of the NHOH group of N-*o*-hydroxyphenylhydroxylamine is the strongest evidence for the formation of complex 4a (Table 4). The 3635 and 3631 cm^−1^ bands, due to the disturbed νOH mode, are ca. −30 cm^−1^ shifted, and the 1364 cm^−1^ band, due to the δOH vibration, is +21 cm^−1^ shifted in relation to the corresponding modes of the SalHy molecule. Some divergence identified for the νOH vibration (Δν_obs_ = −30, Δν_calc_ = −70 cm^−1^) might be due to the strong anharmonicity of this mode. A similar discrepancy was observed for the hydroxylamine-CO [21], *N*-methylhydroxylamine-CO [22], or N-phenylhydroxylamine-CO [14] complexes. The location of other absorptions attributed to SalHy in the 4a structure in the experimental spectra is in accordance with the calculated wavenumbers. The 2144.0 cm^−1^ absorption assigned to the disturbed νCO mode shows a +6 cm^−1^ shift in relation to the carbon monoxide band, which is consistent with the value calculated for the 4a structure (+6 cm^−1^).

### 2.2. The Possible Mechanism of Photodecomposition of SHA

The recorded spectra evidence that the exposure of the C_6_H_4_(OH)CONHOH/Ar (N_2_) matrices to λ = 340 nm radiation (energy of 352 kJ mol^−1^) emitted by the OPO leads to the dissociation of SHA into four pairs of co-products:

C_6_H_4_(OH)CONHOH → C_6_H_4_(OH)NCO + H_2_O (1)

C_6_H_4_(OH)CONHOH → C_6_H_4_(OH)C(O)N + H_2_O (2)

C_6_H_4_(OH)CONHOH → C_6_H_4_(OH)_2_ + HNCO (3)

C_6_H_4_(OH)CONHOH → C_6_H_4_(OH)NHOH + CO (4)

According to literature studies, the photolysis of hydroxamic acids primarily leads to the scission of the N-O bond [10]. The results of our previous studies on the photodissociation of hydroxamic acids as well as the literature data allow us to conclude that, at the beginning of the creation of the C_6_H_4_(OH)NCO + H_2_O pair (1), C_6_H_4_(OH)C(O)N + H_2_O pair (2), and C_6_H_4_(OH)_2_ + HNCO pair (3), there is a fission of the N-O bond that leads to the formation of the C_6_H_4_(OH)CONH and OH radicals:

C_6_H_4_(OH)CONHOH → [C_6_H_4_(OH)CONH]* + OH

The internal energy of the two formed hot radicals may be relatively high (above 300 kJ mol^−1^), meaning that they can undergo further reactions. The identified products suggest two decomposition pathways of the hot [C_6_H_4_(OH)CONH]* radical in the matrix cage in the presence of OH.

Firstly, the radical may donate a hydrogen atom to OH to form the H_2_O molecule and hot nitrene-oxazirine intermediate. The hot intermediate relaxes to the ground state or rearranges to *o*-hydroxyphenyl isocyanate, C_6_H_4_(OH)NCO:

[C_6_H_4_(OH)C(O)NH]* + OH → [C_6_H_4_(OH)C(O)N]* + H_2_O →

C_6_H_4_(OH)C(O)N or C_6_H_4_(OH)NCO and H_2_O

The available data on phenyloxazirene PhNCO rearrangement show that the energy barrier for such a reaction is ca. 61.5 kJ mol^−1^ at the B3LYP/6−31G(d) level [36]. The [C_6_H_4_(OH)C(O)N]* intermediate may be born even with larger amounts of energy, which supports the proposed mechanism for *o*-hydroxyphenyl isocyanate formation. The C_6_H_4_(OH)C(O)N intermediate and the C_6_H_4_(OH)NCO molecule form complexes with the water molecule that is present in the same matrix cage:

C_6_H_4_(OH)C(O)N + H_2_O → C_6_H_4_(OH)C(O)N···H_2_O

C_6_H_4_(OH)NCO + H_2_O → C_6_H_4_(OH)NCO···H_2_O.

Secondly, the [C_6_H_4_(OH)C(O)NH]* radical may decompose into isocyanic acid, HNCO, and C_6_H_4_OH radical. Then, the C_6_H_4_OH radical recombines with OH to form 1,2-dihydroxybenzene, C_6_H_4_(OH)_2_. The C_6_H_4_(OH)_2_ and HNCO molecules trapped in the same cage form molecular complexes, as follows:

[C_6_H_4_(OH)C(O)NH]* + OH → HNCO + C_6_H_4_OH + OH → HNCO + C_6_H_4_(OH)_2_ → C_6_H_4_(OH)_2_···HNCO.

The mechanism of formation of C_6_H_4_(OH)NHOH + CO co-products (4) during SHA photodissociation is similar to that occurring in amide compounds that have been studied both experimentally and theoretically [37,38,39,40,41,42,43]. The ab initio calculations on the photochemistry of benzamide have shed some light on the possible photodissociation channels of this molecule [40]. The mechanisms for the initial relaxation and subsequent dissociation processes have been determined on the basis of the calculated potential energy surfaces and their intersections. It was found that the S_1_/T_1_/T_2_ three-surface intersection determines the photodissociation of benzamide. After excitation to the S_2_ state, the S_2_ → S_1_ internal conversion occurs with a high efficiency, which is explained, among other factors, by a not large energy difference between S_2_ and S_1_, with a value of 63.6 kJ mol^−1^. After relaxation to the S_1_ state, the S_1_ → T_2_ intersystem crossing takes place with a very high rate through the S_1_/T_1_/T_2_ intersection. C-N bond cleavage occurs in the T_2_ state due to the very low barrier (33.5 kJ mol^−1^) for this fission on this pathway. The PhCO radicals (together with the NH_2_ radicals) formed by C-N bond cleavage can further dissociate into CO and Ph. The obtained spectral and calculated data for salicylhydroxamic acid suggest that similar mechanism of photodissociation may occur in SHA, leading to the formation of N-*o*-hydroxyphenylhydroxylamine and carbon monoxide (4). The 340 nm radiation used in the experiment seems sufficient (352 kJ mol^−1^) to induce the transition of SHA to the S_1_ state (according to our calculations, the S_1_ state is ca. 399 kJ mol^−1^ above S_0_), and then intersystem crossing to triplet states may occur. The energy of the excited single and triplet states (S_1_, S_2_, S_3_ and T_1_, T_2_, T_3_, respectively) for SHA electronic transitions were calculated in our study (Table S9). Our results show that the S_1_ level is ca. 18.6 kJ mol^−1^, 60.6 kJ mol^−1^, and 85.5 kJ mol^−1^ above T_3_, T_2_, and T_1_, respectively. Since the calculations indicate that the triplet states are below the S_1_ state, it can be assumed that one of the SHA photolysis paths would proceed similarly to the benzamide molecule according to the following scheme:

C_6_H_4_(OH)CONHOH → C_6_H_4_(OH)CO + NHOH → CO + C_6_H_4_(OH) + NHOH → CO + C_6_H_4_(OH)NHOH.

**Figure 8:**
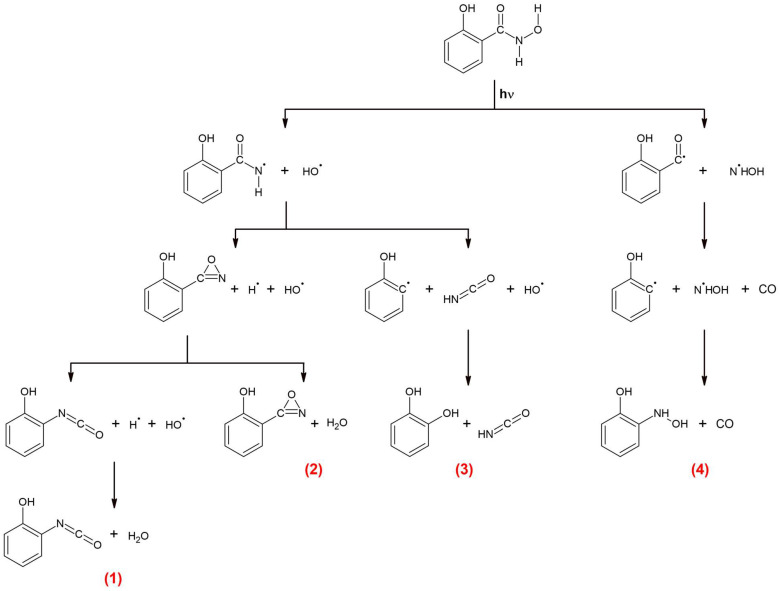
Proposed mechanism of SHA photodecomposition. The numbers (1), (2), (3), (4) refer to the complex designations used in the text.

The first step is the cleavage of the C-N bond and the formation of the C_6_H_4_(OH)CO and NHOH radicals; in the next step, the C_6_H_4_(OH)CO radical dissociates into the *o*-hydroxyphenyl radical and CO. The C_6_H_4_(OH) and NHOH radicals recombine to create C_6_H_4_(OH)NHOH. Finally, the C_6_H_4_(OH)NHOH and CO molecules form a molecular complex. The proposed mechanism of SHA photodecomposition is given in Figure 8.

### 2.3. Other Photolysis Products

In addition to the absorptions characteristic for the four complexes, as discussed above, weak bands due to other photolysis products also appeared in the studied spectra. The absorptions at 1680, 1675, 1630, 1488, 1460, 1417, and 1150 cm^−1^ indicate the formation of salicylaldehyde [44] and those at 1666–1660, 1617, and 1400 cm^−1^ the production of salicylamide [45]. The generation of salicylaldehyde as well as salicylamide may be a result of the *o*-hydroxybenzoyl radical hydrogenation. Amides or anilides are the primary products of the thermolysis of hydroxamic acids [11,19,46], so their formation during photodegradation should also be considered. Identification of CO monomer bands at 2138 and 2136 cm^−1^ may be the effect of the total disintegration of different molecules trapped in matrices. A weak band at 2149 cm^−1^ belonging to the H_2_O-CO complex can also be observed [47]. At ca. 2128 cm^−1^, broad absorption steadily increases for 60 min of irradiation, and the position of this band suggests that it can be attributed to the C=C=O asymmetric stretching mode of the ketoketene molecule. A ketoketene–water complex was observed after the prolonged UV irradiation at λ > 290 nm of salicylic acid in argon matrices. The ν_as_C=C=O band of this complex was observed at 2146 cm^−1^ [48]. In our case, taking into account the precursor structure, ketoketene may be produced together with hydroxylamine, and both molecules may interact forming the C_6_H_4_(=O)=C=O∙∙∙NH_2_OH complex. The anharmonic calculations (Table S10) show that the most intense band of the ketoketene monomer would occur at 2154 cm^−1^ (I_calc_ = 1225 km/mol), and, depending on which group, ketone or ketene would interact as a proton acceptor with hydroxylamine, the ν_as_C=C=O vibration would be shifted towards higher or lower wavenumbers in relation to the monomer band, respectively. Such behavior of this mode was predicted in earlier research for the ketoketene–water complex [48]. The 2155.0 cm^−1^ band that appears in the CO stretching region may be due to the HNCO-CO complex; this complex has been observed previously in the spectrum of photolyzed formohydroxamic acid in argon matrices [21].

## 3. Materials and Methods

The crystalline sample of SHA (98%, Sigma Aldrich, Merck, KGaA, Darmstadt, Germany) was allowed to sublimate at 410 K from a small electric oven assembled inside the vacuum chamber of the cryostat. The temperature of the oven was controlled by the DC-regulated power supply (NDN instruments). The matrices were obtained by the co-deposition of salicylhydroxamic acid vapor with a large excess of argon (nitrogen) into the cold CsI window. The matrix concentration was controlled by the matrix gas flow rate, which was adjusted to minimize the concentration of SHA aggregates and thermolysis products. The low temperature was maintained by means of a closed-cycle helium refrigerator (ARS-2HW, APD-Cryogenics). The FTIR spectra were recorded between 4000 and 500 cm^−1^ in a transmission mode by means of a Nicolet iS50 FTIR spectrometer with a resolution of 0.5 cm^−1^, using a liquid N_2_-cooled MCT detector. Photochemical reactions were induced in the SHA/Ar (N_2_) matrices by UV radiation of a pulsed (7 ns) optical parametric oscillator Vibrant 355 (Opotek, Inc., Carlsbad, CA, USA) (repetition rate 10 Hz, average pulse energies ~7.0 mJ (325 nm) and ~2.5 mJ (250 nm)), pumped with a pulsed Nd:YAG laser (Quantel, Bozeman, MT, USA). The experiments started using λ = 400 nm light and proceeded with a gradual decrease in the output wavelength. The process was controlled by recording the infrared spectra of the matrix after each irradiation. The best experimental conditions for the observation of SHA photochemistry were obtained by irradiation with a wavelength of λ = 340 nm.

All the calculations were performed with the Gaussian 16 program [49]. The structures of the photoproducts were optimized at the DFT B3LYPD3/6-311++G(2d,2p) level [50,51], and GD3 dispersion correction was used. The literature reports that, despite its popularity, the global hybrid functional B3LYP has its limitations (errors in the potential energy surface and in the harmonic approximation used) [52]. However, B3LYP (or rather, its dispersion-corrected versions) is functional, with a proven usefulness for the computation of IR spectra [53,54]. Taking into account our experience with DFT calculations for small organic compounds and literature studies on the applicability of various functionals, we chose B3LYPD3 to simulate the IR spectra of photoproducts and compare them with experimental spectra. The force-constant matrices were calculated at the same level for the precursor and the photoproducts to evaluate the harmonic frequencies and zero-point vibrational (ZPE) corrections. The structures of the complexes were also optimized at the B3LYPD3 level, and their binding energies (ΔE^CP^) were corrected by means of the Boys–Bernardi full-counterpoise procedure [55]. The selected structural parameters calculated for all the optimized complexes are collected in Tables S11–S14 in the Supplementary Materials. Anharmonic wavenumbers were calculated for the monomeric species at the same level of theory. The vertical excitation energies were calculated using time-dependent density functional theory, TD-DFT [56,57].

## 4. Conclusions

Laser photolysis of salicylhydroxamic acid (SHA), which is a compound important from the point of view of biology and medicine, in two low-temperature matrices was performed for the first time. After irradiation with a 340 nm laser line, the formation of new isocyanate complexes, previously unpublished in the literature, was observed: C_6_H_4_(OH)NCO⋯H_2_O (1), C_6_H_4_(OH)C(O)N⋯H_2_O (2), and C_6_H_4_(OH)_2_⋯HNCO (3), for which spectroscopic characteristics in the infrared range were obtained. The N-*o*-hydroxyphenylhydroxylamine molecule was identified for the first time as the co-product of the SHA photodissociation reaction: C_6_H_4_(OH)CONHOH → C_6_H_4_(OH)NHOH + CO (4). UV radiation-induced photodecomposition reaction channels were determined for SHA. It was demonstrated that, for the studied molecule, one of the two reaction channels is the scission of the N-O bond (creating complexes (1), (2), and (3)), while the second path leads to C-N bond breaking (leading to the creation of complex (4)). In both the studied matrices, photochemical reactions occurred according to the same mechanism, but a clear influence of the nitrogen matrix on the formation of specific structures of the isocyanic acid–1,2-dihydroxybenzene complex was observed: two structures were identified in the Ar matrix and one structure in the N_2_ matrix. The B3LYPD3 computational method with the 6-311++G(2d,2p) basis set was used to optimize the structures of the four complexes produced throughout SHA photolysis. Four stable structures were obtained for the C_6_H_4_(OH)NCO⋯H_2_O and C_6_H_4_(OH)_2_⋯HNCO complexes, and three configurations were found for the C_6_H_4_(OH)C(O)N⋯H_2_O and C_6_H_4_(OH)NHOH⋯CO pairs. The spectra evidenced that, during SHA photodissociation, structures corresponding to the global minimum as well as those due to the local minima are formed.

## Figures and Tables

**Table 1:** Wavenumbers and wavenumbers shifts (in cm^−1^) calculated and observed for the C_6_H_4_(OH)NCO-H_2_O complex in solid argon and nitrogen ^a^.

Experimental							Calculated			Assignment
Monomer	1a		1b		1c		1a	1b	1c	
ν	ν	Δν_exp_	ν	Δν_exp_	ν	Δν_exp_	Δν_cal_	Δν_cal_	Δν_cal_	
3734.0 ^b^ [3727.0]	3728.0 [3720.0]	−8	3719.0 [3715.0]	−15	3724.0	−10	−8 (81)	−25 (131)	−13 (94)	ν_as_OH (ν_3_ H_2_O)
3638.0 ^b^ [3634.0]	-	-	3567 br [3564]	−71	3624	−14	−7 (10)	−66 (244)	−17 (18)	ν_s_OH (ν_1_ H_2_O)
3582	3365.5 3352.0 3345.0 [3364 3349]	−227	3574 [3575]	−8	-	-	−194 (736)	−2 (65)	6 (72)	νOH
2289 ^b^	2285.0 [2283.0]	−4	2280.0 [2283.0]	−9	2289.0	0	1 (1577)	−2 (1684)	4 (1660)	ν_as_NCO
1589.0 ^b^ [1597.0]	1588.0	−1	1599.0	10	1593.0	4	−2 (98)	15 (44)	2 (132)	δOH (ν_2_ H_2_O)
1589	1584.5 [1584 sh]	−4.5	1591.0 [1591 sh]	2	1589.0	0	−4 (66)	3 (56)	1 (55)	νC=C
1516	1526.0 [1529]	10	1526.0 [1529]	10	1526.0	10	0 (123)	3 (124)	1 (105)	δCH
1345	1344 [1344]	1	-	-	-	-	7 (70)	0 (24)	3 (30)	δCH
1294	1306.5 1304.0 [1304]	12.5	1299.0 [1296]	5	1295.0	1	10 (82)	4 (35)	0 (42)	νC=C
1249	1255.5 [1255]	6.5	1247.0 [1244]	−2	1254 sh	5	5 (52)	−8 (86)	7 (59)	νC-O
1200	1203.0 [1200]	3	1203.0 [1200]	3	1202 sh	2	12 (183)	9 (173)	0 (170)	δOH
750	750.0 749.0 [752]	0	753.0 [756]	3	759.0	9	1 (34)	5 (81)	9 (68)	γCH
417	698 [697]	281	-	-	-	-	274 (134)	5 (86)	18 (94)	γOH

^a^ In all calculations, the B3LYPD3/6-311++G(2d,2p) method was used. The calculated intensities are given in parentheses (in km mol^−1^). The wavenumbers from the nitrogen matrix are given in brackets; ^b^ the experimental wavenumbers for the monomers were taken from refs. [30,31]; for the C_6_H_4_(OH)NCO monomer, anharmonic wavenumbers were used for the Δν_exp_ calculations (Table S2). br = broad band; sh = shoulder.

**Table 2:** Wavenumbers and wavenumbers shifts (in cm^−1^) calculated and observed for the C_6_H_4_(OH)C(O)N-H_2_O complex in solid argon and nitrogen ^a^.

Experimental			Calculated			Assignment
Monomer	2a		2a	2b	2c	
ν	ν	Δν_exp_	Δν_cal_	Δν_cal_	Δν_cal_	
3734.0 ^b^ [3727.0]	3703	−31	−41 (113)	−25 (72)	−21 (138)	ν_as_OH (ν_3_ H_2_O)
3638.0 ^b^ [3634.0]	3537 [3535]	−101	−151 (294)	−13 (21)	−52 (147)	ν_s_OH (ν_1_ H_2_O)
3513	3208 br [3218 br]	−310	−323 (1027)	−13 (139)	−18 (159)	νOH
1737	1793 1789 [1790]	56	5 (290)	−23 (373)	0 (310)	ν_as_CCN
1589.0 ^b^ [1597.0]	1587	−2	−3 (147)	12 (30)	9 (107)	δOH (ν_2_ H_2_O)
1485	1481 [1480]	−4	−1 (118)	3 (75)	8 (84)	δCH
1225	1218 [1217]	−7	−2 (117)	2 (34)	−10 (22)	νC-O

^a^ In all calculations, the B3LYPD3/6-311++G(2d,2p) method was used. The calculated intensities are given in parentheses (in km mol^−1^). The wavenumbers from the nitrogen matrix are given in brackets; ^b^ the experimental wavenumbers for the H_2_O monomer were taken from ref. [30]; for the C_6_H_4_(OH)C(O)N monomer, anharmonic wavenumbers were used for the Δν_exp_ calculations (Table S4). br = broad band.

**Table 3:** Wavenumbers and wavenumbers shifts (in cm^−1^) calculated and observed for the C_6_H_4_(OH)_2_-HNCO complex in solid argon and nitrogen ^a^.

Experimental					Calculated			Assignment
Monomer	3b		3c		3a	3b	3c	
ν	ν	Δν_exp_	ν	Δν_exp_	Δν_cal_	Δν_cal_	Δν_cal_	
3649 ^b^	3643.0	−6	3463 br 3455.0 [3460 br]	−186	−64 (111)	−1 (90)	−183 (765)	νOH
3596 ^b^	3585.0	−11	3589.0	−7	−37 (109)	−10 (107)	−8 (92)	νOH
3517 ^b^ [3510]	3429.0 3425.5 3412.0 3389.0 3382.0	−105	3497 br [3492 br]	−20	−87 (215)	−185 (1175)	−41 (162)	νNH
2259 ^b^ [2265]	2258.0	−1	2252.0 [2255.0]	−7	−12 (538)	0 (931)	−2 (925)	ν_as_NCO
1545	1547.0	2	1547.0 [1548.0]	2	−5 (117)	2 (108)	3 (100)	δCH
1264	1263.0	−1	1260.0 [1259.0]	−4	−1 (147)	0 (113)	−1 (144)	νC-O
1239	1237 br	−2	1242.0 [1240.0]	3	20 (74)	−4 (215)	10 (110)	νC-O
1194 ^b^	-	-	1210.0 [1211.0]	16	13 (85)	4 (25)	10 (93)	δOH
1149 ^b^	1150.0	1	1160.0 [1159.0]	11	22 (94)	2 (67)	19 (90)	δOH
770 ^b^ [770]	836.0	66	850.0 [850.0]	80	35 (223)	62 (275)	96 (278)	δNH
752	753.0	1	755.0 [756.0]	3	20 (58)	6 (69)	3 (76)	γCH

^a^ In all calculations, the B3LYPD3/6-311++G(2d,2p) method was used. The calculated intensities are given in parentheses (in km mol^−1^). The wavenumbers from the nitrogen matrix are given in brackets; ^b^ the experimental wavenumbers for the monomers were taken from refs. [26,27,32]; for the C_6_H_4_(OH)_2_ monomer, some anharmonic wavenumbers were used for the Δν_exp_ calculations (Table S6). br = broad band.

**Table 4:** Wavenumbers and wavenumbers shifts (in cm^−1^) calculated and observed for the C_6_H_4_(OH)NHOH-CO complex in solid argon and nitrogen ^a^.

Experimental			Calculated			Assignment
Monomer	4a		4a	4b	4c	
ν	ν	Δν_exp_	Δν_cal_	Δν_cal_	Δν_cal_	
3629	-	-	0 (70)	−68 (410)	0 (70)	νOH
3661	3635.0 3631.0 [3630 br]	−30	−70 (145)	1 (70)	0 (75)	ν(N)OH
2138 ^b^ [2140]	2144.0 [2146]	6	6 (88)	22 (95)	−6 (59)	νC≡O
1500	1498.0 [1500]	−2	0 (71)	2 (67)	1 (70)	δCH
1343	1364.0	21	27 (97)	1 (80)	2 (90)	δNOH
1329	-	-	3 (30)	6 (63)	2 (36)	δOH
1249	1255 [1255 sh]	6	1 (100)	2 (92)	2 (94)	νC-O
877	871.0	−6	−9 (70)	2 (90)	1 (98)	γNH

^a^ In all calculations, the B3LYPD3/6-311++G(2d,2p) method was used. The calculated intensities are given in parentheses (in km mol^−1^). The wavenumbers from nitrogen matrix are given in brackets; ^b^ the experimental wavenumber for the CO monomer was taken from refs. [28,29]; for the C_6_H_4_(OH)NHOH monomer, anharmonic wavenumbers were used for the Δν_exp_ calculations (Table S8). br = broad band; sh = shoulder.

## Data Availability

The data presented in this study are available in this article.

## Supplementary Materials

The following supporting information can be downloaded at https://www.mdpi.com/article/10.3390/molecules29040862/s1: Figure S1: 2322–1775 cm^−1^ region in the SHA/Ar matrix during irradiation at wavelengths between 370 and 350 nm; Table S1: Wavenumbers and wavenumbers shifts (in cm^−1^) calculated for the C_6_H_4_(OH)NCO-H_2_O complexes at the B3LYPD3/6-311++G(2d,2p) level of theory. The IR-calculated intensities in km mol^−1^; Table S2: Anharmonic and harmonic wavenumbers (in cm^−1^) calculated for the C_6_H_4_(OH)NCO molecule at the B3LYPD3/6-311++G(2d,2p) level of theory. The IR-calculated intensities expressed in km mol^−1^; Table S3: Wavenumbers and wavenumbers shifts (in cm^−1^) calculated for the C_6_H_4_(OH)C(O)N-H_2_O complexes at the B3LYPD3/6-311++G(2d,2p) level of theory. The IR-calculated intensities in km mol^−1^; Table S4: Anharmonic and harmonic wavenumbers (in cm^−1^) calculated for the C_6_H_4_(OH)C(O)N molecule at the B3LYPD3/6-311++G(2d,2p) level of theory. The IR-calculated intensities expressed in km mol^−1^; Table S5: Wavenumbers and wavenumbers shifts (in cm^−1^) calculated for the C_6_H_4_(OH)_2_-HNCO complexes at the B3LYPD3/6-311++G(2d,2p) level of theory. The IR-calculated intensities in km mol^−1^; Table S6: Anharmonic and harmonic wavenumbers (in cm^−1^) calculated for the C_6_H_4_(OH)_2_ molecule at the B3LYPD3/6-311++G(2d,2p) level of theory. The IR-calculated intensities expressed in km mol^−1^; Table S7: Wavenumbers and wavenumbers shifts (in cm^−1^) calculated for the C_6_H_4_(OH)NHOH-CO complexes at the B3LYPD3/6-311++G(2d,2p) level of theory. The IR-calculated intensities in km mol^−1^; Table S8: Anharmonic and harmonic wavenumbers (in cm^−1^) calculated for the C_6_H_4_(OH)NHOH molecule at the B3LYPD3/6-311++G(2d,2p) level of theory. The IR-calculated intensities expressed in km mol^−1^; Table S9: Energy of vertical excitations ΔE (nm) and corresponding oscillator strength (f) calculated using the TD-DFT (B3LYP/6-311++G(2d,2p)) method; Table S10: Anharmonic and harmonic wavenumbers (in cm^−1^) calculated for the ketoketene C_6_H_4_(=O)=C=O molecule at the B3LYP/6-311++G(2d,2p) level of theory. The IR-calculated intensities expressed in km mol^−1^; Table S11: Selected structural parameters*^a^* calculated for the C_6_H_4_(OH)NCO-H_2_O complexes at the B3LYPD3/6-311++G(2d,2p) level of theory; Table S12: Selected structural parameters*^a^* calculated for the C_6_H_4_(OH)C(O)N-H_2_O complexes at the B3LYPD3/6-311++G(2d,2p) level of theory; Table S13: Selected structural parameters*^a^* calculated for the C_6_H_4_(OH)_2_-HNCO complexes at the B3LYPD3/6-311++G(2d,2p) level of theory; Table S14 Selected structural parameters calculated for the C_6_H_4_(OH)NHOH-CO complexes at the B3LYPD3/6-311++G(2d,2p) level of theory.
