# Supplementary material for: UV Laser-Induced Photodecomposition of Matrix-Isolated Salicylhydroxamic Acid: Identification of New Isocyanate Complexes
Source: Molecules. 2024 Feb 15;29(4):862. doi: 10.3390/molecules29040862 (PMC10892988; doi:10.3390/molecules29040862)

Supplementary data

UV laser induced photodecomposition of matrix isolated salicylhydroxamic acid: identification of new isocyanate complexes

Magdalena Sałdyka*, Zofia Mielke*

Faculty of Chemistry, University of Wroclaw, F. Joliot-Curie 14, 50-383 Wroclaw, Poland

Figure S1. 2322-1775 cm^{-1} region in the SHA/Ar matrix during irradiation at wavelengths between 370 and 350 nm

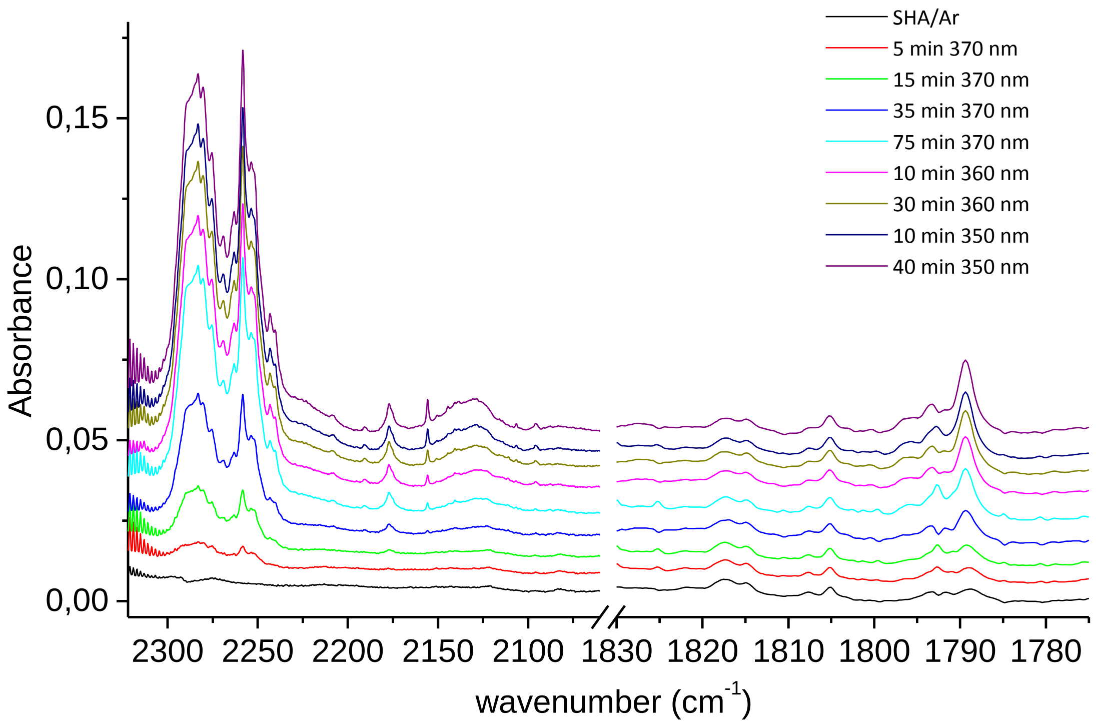

Table S1 Wavenumbers and wavenumbers shifts (in cm^{-1}) calculated for the $C_6H_4(OH)NCO-H_2O$ complexes at the B3LYPD3/6-311++G(2d,2p) level of theory. The IR calculated intensities in km mol^{-1}.

**1a**	**Int.**	**Δν**	**1b**	**Int.**	**Δν**	**1c**	**Int.**	**Δν**	**1d**	**Int.**	**Δν**	**Assignment**
3915	81	-8	3898	131	-25	3910	94	-13	3920	69	-3	ν_{as}OH (H_2O)
3814	10	-7	3755	244	-66	3804	18	-17	3818	13	-3	ν_sOH (H_2O)
3576	736	-194	3768	65	-2	3776	72	6	3774	89	4	νOH
3203	6	-2	3204	7	-1	3204	10	-1	3215	1	10	νCH
3196	10	-2	3197	1	-1	3197	5	-1	3203	10	5	νCH
3180	8	-2	3185	8	3	3188	6	6	3190	6	8	νCH
3172	0	-2	3176	1	2	3176	7	2	3174	8	0	νCH
2332	1577	1	2329	1684	-2	2335	1660	4	2330	1682	-1	ν_{as}NCO
1642	14	-4	1645	7	-1	1646	1	0	1644	0	-2	νC=C, δOH
1637	98	-2	1654	44	15	1641	132	2	1640	83	1	δOH (H_2O)
1621	66	-4	1628	56	3	1626	55	1	1623	53	-2	νC=C, δOH
1547	123	0	1550	124	3	1548	105	1	1543	125	-4	νC-N, δCH
1501	6	0	1501	24	0	1500	10	-1	1500	10	-1	δCH
1474	16	2	1473	13	1	1473	8	1	1469	13	-3	ν_sNCO
1382	70	7	1375	24	0	1378	30	3	1373	28	-2	δCH
1331	82	10	1325	35	4	1321	42	0	1320	41	-1	νC=C
1283	52	5	1270	86	-8	1285	59	7	1275	71	-2	νC-O
1236	183	12	1233	173	9	1224	170	0	1224	154	0	δOH
1191	4	-1	1196	2	4	1193	7	3	1189	14	0	δCH
1165	16	0	1173	11	8	1163	15	-1	1164	13	0	δCH
1108	36	5	1103	20	0	1102	21	-1	1100	25	-3	δCC
1058	19	3	1058	19	3	1054	16	2	1051	17	-5	δCH
983	0	-3	996	0	10	993	0	6	1008	0	22	γCH
943	3	-4	954	3	7	966	4	20	952	3	1	γCH
865	0	1	876	1	12	875	0	11	880	2	16	γCH
858	10	7	848	14	-3	851	14	0	851	11	0	δCC
757	34	1	761	81	5	765	68	9	765	72	9	γCH
751	22	-7	758	32	0	750	28	-5	753	26	-5	δCC
742	15	6	737	0	1	742	2	4	744	0	8	γCC
708	134	274	439	86	5	452	94	18	442	85	8	γOH
655	27	-7	662	22	0	657	27	-3	659	26	-3	δNCO
583	0	3	579	2	-1	578	0	1	578	0	1	δCC
566	16	-2	570	20	2	565	22	-4	567	16	-1	γNCO
560	0	-1	561	2	0	560	3	-1	564	4	3	γCC

540	6	14	534	58	8	520	8	-1	520	10	-1	δCCO
464	2	-5	464	0	-5	468	5	2	468	26	2	γCH
448	2	3	442	8	-3	441	5	0	441	5	0	δCC
288	2		299	3		288	1		288	1		Skeleton def
275	7		290	10		285	5		284	12		Skeleton def
186	16		193	2		193	0		197	0		Skeleton def
90	5		90	20		88	56		87	0		Skeleton def
55	3		60	12		62	2		55	11		Skeleton def
350	22		500	112		316	169		118	108		Intermolecular
241	204		268	96		222	81		110	8		Intermolecular
152	7		131	10		145	8		103	32		Intermolecular
110	47		114	84		90	28		95	17		Intermolecular
64	1		62	8		73	32		36	1		Intermolecular
24	7		22	1		21	3		6	10		Intermolecular

Table S2 Anharmonic and harmonic wavenumbers (in cm^{-1}) calculated for the C_6H_4(OH)NCO molecule at the B3LYPD3/6-311++G(2d,2p) level of theory. The IR calculated intensities expressed in km mol^{-1}.

Fundamental Bands

Mode(n)	E(harm)	E(anharm)	I(harm)	I(anharm)
1(1)	3772.811	3582.349	90.21511503	75.80957453
2(1)	3205.213	3074.573	4.57236032	6.65531635
3(1)	3197.421	3051.788	8.29877541	9.13279681
4(1)	3182.965	3085.077	6.46848422	4.96477617
5(1)	3175.082	3024.919	0.65349036	6.30031072
6(1)	2329.818	2292.889	1659.78552243	857.54649771
7(1)	1645.494	1604.704	4.65398847	6.83479988
8(1)	1625.635	1588.631	53.35526227	34.14319417
9(1)	1546.918	1515.600	123.71333558	76.71766708
10(1)	1501.837	1475.960	10.41367439	5.72596596
11(1)	1471.200	1445.823	12.68585611	7.56987907
12(1)	1374.630	1345.047	25.12200468	4.19333168
13(1)	1321.398	1293.836	50.94659653	38.39906481
14(1)	1278.600	1249.192	70.14341128	48.66466720
15(1)	1224.586	1200.254	155.96119823	105.49055703
16(1)	1191.442	1170.013	5.98683665	1.64077078
17(1)	1164.876	1154.503	15.71332106	19.70336607
18(1)	1103.201	1081.390	21.49556735	18.50285667
19(1)	1052.771	1034.307	17.69826432	11.92577159
20(1)	985.640	986.693	0.11786964	0.02335446
21(1)	944.451	944.066	3.18487089	3.16116160
22(1)	864.845	855.744	0.75859168	0.10794109
23(1)	851.919	833.225	10.64722612	3.75802451
24(1)	756.144	749.545	71.31569542	46.30827381
25(1)	755.860	750.168	26.29012434	24.85094480
26(1)	738.616	731.125	0.32672763	5.56041442
27(1)	660.572	655.499	26.14769837	18.03032349
28(1)	578.770	577.921	0.39081442	0.34520203
29(1)	569.785	567.777	19.32484229	9.71005085
30(1)	561.030	559.345	1.64303993	1.39725772
31(1)	520.496	514.256	8.00389461	6.16799413
32(1)	465.822	579.993	18.08657393	10.65866190
33(1)	443.273	416.860	78.58103057	67.71001903
34(1)	441.731	435.444	5.58703548	5.02976071
35(1)	287.791	285.151	0.98026020	0.82429332
36(1)	283.981	281.418	11.71341798	11.45488136
37(1)	192.082	190.535	0.01171187	0.01148930
38(1)	91.014	88.809	2.34898898	2.29186602
39(1)	58.932	61.848	0.20953462	0.42573772

Table S3 Wavenumbers and wavenumbers shifts (in cm^{-1}) calculated for the $C_6H_4(OH)C(O)N-H_2O$ complexes at the B3LYPD3/6-311++G(2d,2p) level of theory. The IR calculated intensities in km mol^{-1}.

2a	Int.	Δν	2b	Int.	Δν	2c	Int.	Δν	Assignment
3882	113	-41	3898	72	-25	3902	138	-21	ν_{as}OH (H_2O)
3670	294	-151	3808	21	-13	3769	147	-52	ν_{s}OH (H_2O)
3399	1027	-323	3709	139	-13	3704	159	-18	νOH
3207	5	-1	3207	9	-1	3207	11	-1	νCH
3202	6	-1	3202	4	-1	3202	4	-1	νCH
3181	4	-3	3190	14	3	3185	6	1	νCH
3176	2	-2	3177	7	2	3179	1	1	νCH
1775	290	5	1747	373	-23	1770	310	0	ν_{as}CCN
1654	60	-4	1658	91	-1	1660	23	2	νC=C, δOH
1636	147	-3	1651	30	12	1648	107	9	δOH (H_2O)
1601	42	-13	1612	57	-2	1616	50	2	νC=C, δOH
1520	118	-1	1524	75	3	1526	84	8	δCH
1508	1	-1	1508	40	-1	1508	50	-1	δCH
1414	91	28	1392	31	6	1391	19	5	δCH, δOH
1355	36	3	1352	28	0	1353	26	1	νC=C, δCH
1293	84	43	1250	165	0	1256	186	6	δOH, δCH
1275	117	-2	1279	34	2	1267	22	-10	νC-O
1232	12	4	1235	0	7	1236	2	6	ν_{as}CCO
1193	14	1	1194	5	2	1200	10	8	δCH
1145	44	-15	1166	82	6	1164	47	4	δCH, ν_{s}CCO
1129	1	7	1122	4	0	1123	1	1	δCH
1053	16	3	1052	16	2	1055	24	5	δCH
1006	0	0	1017	0	11	1016	1	10	γCH
973	1	1	998	2	25	978	1	6	γCH
885	2	5	888	1	8	895	3	15	γCH
870	10	10	860	7	0	856	10	-4	δCCC
799	97	329	484	80	14	452	159	-18	γOH
772	87	-1	782	52	9	778	44	5	γCH
760	19	8	756	4	4	744	10	-8	γCH
730	10	-6	733	12	-3	736	9	0	δCCC, δNCO
633	15	3	640	29	10	630	30	0	γCC
592	19	7	580	10	-5	584	18	-1	δCCC, δNCO
570	2	9	563	0	3	564	2	4	δCCO

536	16	11	530	12	5	525	17	0	γCH
522	9	15	494	5	-13	510	13	3	δCCN
440	15	28	415	20	3	414	17	2	γCH
408	14	44	420	1	8	408	8	44	δCCO
342	6	3	344	42	5	342	3	3	Skeleton def
240	18	10	230	0	0	230	4	0	Skeleton def
220	15	43	203	3	26	192	0	15	Skeleton def
117	1	-7	116	17	0	126	1	2	Skeleton def
65	0	15	56	25	6	46	0	-4	Skeleton def
509	85		312	476		444	39		Intermolecular
365	42		256	2		281	112		Intermolecular
281	128		138	29		121	15		Intermolecular
191	16		104	25		97	94		Intermolecular
146	2		95	6		73	34		Intermolecular
46	7		34	0		23	0		Intermolecular

Table S4 Anharmonic and harmonic wavenumbers (in cm^{-1}) calculated for the $C_6H_4(OH)C(O)N$ molecule at the B3LYPD3/6-311++G(2d,2p) level of theory. The IR calculated intensities expressed in km mol^{-1}.

```
Fundamental Bands
 -----------------
   Mode(n)                    E(harm)   E(anharm)        I(harm)       I(anharm)
      1(1)                   3721.929    3513.027   129.69583062    126.07211398
      2(1)                   3208.418    3077.650     3.39340087      4.86885705
      3(1)                   3202.896    3053.763     4.40620307      1.75631116
      4(1)                   3183.954    3053.175     3.20046076      3.97062932
      5(1)                   3178.244    3014.430     0.75937044      2.33766589
      6(1)                   1769.722    1737.493   311.56441466    155.39777193
      7(1)                   1657.692    1616.188    67.97701199     42.69642433
      8(1)                   1613.530    1573.558    50.77266085     22.47726866
      9(1)                   1518.878    1485.483    71.68526073     47.78085580
     10(1)                   1509.347    1477.673    48.27395204     38.94465511
     11(1)                   1386.193    1355.186    23.31324296     11.10465136
     12(1)                   1351.931    1319.638    28.46973743     32.60392333
     13(1)                   1276.659    1251.469    33.15346237     23.45676637
     14(1)                   1249.838    1225.393   151.52617021     93.84894079
     15(1)                   1227.715    1204.513    11.68781396      1.46248982
     16(1)                   1192.547    1172.439     6.96971968      9.87609693
     17(1)                   1159.999    1133.989    67.70433895     47.92166069
     18(1)                   1122.257    1104.568     1.83501828      2.49680894
     19(1)                   1049.616    1028.602    17.43639731     14.84864361
     20(1)                   1005.521     998.640     0.29125019      0.24880044
     21(1)                    972.285     959.961     0.97505602      0.31091134
     22(1)                    880.316     876.422     2.46143053      2.56959677
     23(1)                    859.762     847.926     8.20318733      8.30634844
     24(1)                    773.111     762.411    46.13461963     49.76053628
     25(1)                    751.756     792.073     7.67731646      0.02287167
     26(1)                    735.670     726.728     7.41246395      5.24648539
     27(1)                    629.640     628.539    30.54683953     28.29466561
     28(1)                    584.687     579.957    14.68051981     10.38799004
     29(1)                    560.625     556.123     2.45285574      1.52148317
     30(1)                    524.903     513.651    15.77745631     59.97985956
     31(1)                    507.696     504.278    10.98408887      8.73330282
     32(1)                    470.103     541.557    78.13147380     11.86029329
     33(1)                    412.206     411.213     1.19268986      5.04623045
     34(1)                    409.286     407.123     2.86183349      2.35626430
     35(1)                    339.113     331.696     4.46732187      2.79688870
     36(1)                    230.951     234.852     0.00019406      0.00707475
     37(1)                    176.580     175.084     5.87563770      5.76192946
```

```
38(1)                    124.546    128.884      1.56061147       0.77949514
39(1)                     49.059     85.590      0.17094330       0.38018930
```

Table S5 Wavenumbers and wavenumbers shifts (in cm^{-1}) calculated for the $C_6H_4(OH)_2$-HNCO complexes at the B3LYPD3/6-311++G(2d,2p) level of theory. The IR calculated intensities in km mol^{-1}.

3a	Int.	Δν	3b	Int.	Δν	3c	Int.	Δν	3d	Int.	Δν	Assignment
3788	111	-64	3851	90	-1	3669	765	-183	3840	86	-12	νOH
3763	109	-37	3790	107	-10	3792	92	-8	3809	86	9	νOH
3596	215	-87	3498	1175	-185	3643	162	-41	3548	503	-135	νNH (HNCO)
3203	4	1	3205	5	3	3200	7	-2	3206	3	4	νCH
3196	6	3	3195	9	2	3190	14	-3	3197	7	4	νCH
3182	7	3	3183	3	4	3178	9	-1	3183	4	4	νCH
3175	0	20	3160	9	5	3169	3	4	3160	7	10	νCH
2303	538	-12	2315	931	0	2313	925	-2	2312	618	-3	ν_{as}NCO (HNCO)
1645	4	-5	1656	23	6	1651	23	1	1648	13	-2	νC=C, δOH
1632	43	-12	1644	34	0	1641	40	-3	1646	13	4	νC=C, δOH
1580	117	-5	1587	108	2	1588	100	3	1583	97	-2	δCH, δCC
1504	10	-4	1510	30	2	1509	42	1	1508	24	0	δCH
1392	31	0	1392	48	0	1397	51	5	1383	23	-9	δCH, δOH
1348	26	-14	1362	44	0	1380	68	18	1358	34	-4	δCH, δOH
1322	5	-4	1330	10	4	1322	2	-4	1327	2	1	ν_sNCO (HNCO)
1293	147	-1	1294	113	0	1293	144	-1	1295	167	1	ν_sC-O
1285	74	20	1261	215	-4	1275	110	10	1256	72	-9	ν_{as}C-O
1219	85	13	1213	25	4	1216	93	10	1203	51	-3	δOH, δCH
1186	3	-1	1191	7	4	1186	2	-1	1189	13	2	δCH
1193	94	22	1173	67	2	1190	90	19	1173	78	2	δOH, δCH
1115	32	4	1112	70	1	1118	30	7	1112	74	1	δCC, δOH
1048	12	-4	1055	12	3	1052	16	0	1053	12	1	δCC
988	0	21	979	0	12	966	0	-1	982	0	15	γCH
948	4	30	932	6	14	924	15	6	934	4	16	γCH
849	223	35	875	275	62	910	278	96	904	215	90	δNH (HNCO)
869	20	7	860	51	2	865	29	3	860	5	-2	δCC
868	16	2	850	33	-16	852	1	-14	855	0	-11	γCH
782	20	5	780	24	3	781	20	4	775	23	-2	δCC
764	58	20	750	69	6	746	76	3	750	77	6	γCH
743	6	10	735	4	2	740	2	7	736	1	3	Skeleton def
631	3	-5	676	10	40	664	50	28	652	0	16	γNCO (HNCO)
592	35	14	636	20	58	632	4	54	626	18	48	δNCO (HNCO)

590	9	-1	591	5	0	591	2	0	591	3	0	δCC
575	0	5	570	1	0	572	1	2	570	0	0	Skeleton def
562	8	5	561	14	4	563	6	6	556	3	-1	δCCO
522	110	107	406	37	-9	534	19	119	481	31	66	γOH
460	23	3	459	3	2	459	5	2	458	3	1	Skeleton def
453	0	2	446	10	-5	456	5	4	446	19	-5	δCCC
315	1	5	323	84	13	330	1	20	307	18	-3	δCCO
300	2	8	293	1	1	293	1	1	292	10	0	Skeleton def
286	127	83	207	82	4	425	107	222	373	223	170	γOH
197	8	14	188	50	5	189	3	6	200	1	17	Skeleton def
258	44		516	105		182	98		251	19		Intermolecular
128	5		132	7		130	2		142	2		Intermolecular
113	7		66	0		57	4		75	0		Intermolecular
78	0		32	1		51	4		41	1		Intermolecular
50	0		12	0		18	1		26	0		Intermolecular
34	0		8	0		9	2		11	0		Intermolecular

Table S6 Anharmonic and harmonic wavenumbers (in cm^{-1}) calculated for the $C_6H_4(OH)_2$ molecule at the B3LYPD3/6-311++G(2d,2p) level of theory. The IR calculated intensities expressed in km mol^{-1}.

```
Fundamental Bands
 -----------------
   Mode(n)                    E(harm)   E(anharm)       I(harm)        I(anharm)
      1(1)                   3852.181    3664.150     80.34842844      64.85368609
      2(1)                   3801.275    3601.891     98.16540286      89.56714515
      3(1)                   3202.605    3096.758      6.21929727       3.05754420
      4(1)                   3193.080    3059.103     11.16799423       5.91360651
      5(1)                   3179.155    3033.327      4.31477759      21.56923944
      6(1)                   3155.745    3012.414     10.87596395       1.86964995
      7(1)                   1650.180    1605.558     19.72891124       7.06005894
      8(1)                   1644.044    1602.536     28.77701161      17.55036674
      9(1)                   1585.679    1545.405    106.77154517      95.17734764
     10(1)                   1508.601    1472.661     31.92221100      13.60845166
     11(1)                   1392.806    1358.940     32.66429519      31.10442879
     12(1)                   1362.418    1328.901     50.47165742      80.57984241
     13(1)                   1294.522    1263.650    165.69437014      95.23678244
     14(1)                   1265.270    1239.398    112.45080829      79.45278768
     15(1)                   1208.640    1204.494     52.00156435      79.62462997
     16(1)                   1187.792    1166.728      6.22315862       8.44890992
     17(1)                   1171.746    1146.476     78.27114036     101.32069107
     18(1)                   1111.852    1071.467     70.03670677       6.04272225
     19(1)                   1052.716    1034.673     13.29695657      10.59548995
     20(1)                    967.389    1007.441      0.15634881       0.10240744
     21(1)                    918.217     956.511      4.37684842       4.60499053
     22(1)                    862.766     854.247     22.55140679      17.88563348
     23(1)                    846.226     861.681      0.13395143       0.14859589
     24(1)                    777.909     767.853     21.22580858      20.30939801
     25(1)                    766.457     751.504     75.03238788      20.02887786
     26(1)                    733.834     771.379      5.63408258       1.21541534
     27(1)                    591.752     582.517      2.63947275       2.46607826
     28(1)                    569.170     587.473      0.33969059       0.05164076
     29(1)                    557.677     551.998      8.64655725       7.15101639
     30(1)                    457.986     508.399      0.05125931      45.92015833
     31(1)                    450.800     448.186      7.17526252       7.16477018
     32(1)                    415.341     391.181     72.56960559      83.56908977
     33(1)                    310.274     313.264      4.80037342       2.93661156
     34(1)                    292.446     300.618      0.40417264       3.13311997
     35(1)                    203.370     251.281    105.04222722      64.35570276
     36(1)                    183.118     216.881     31.38903166      31.12237373
```

Table S7 Wavenumbers and wavenumbers shifts (in cm^{-1}) calculated for the $C_6H_4(OH)NHOH$-CO complexes at the B3LYPD3/6-311++G(2d,2p) level of theory. The IR calculated intensities in km mol^{-1}.

4a	Int.	Δν	4b	Int.	Δν	4c	Int.	Δν	Assignment
3845	70	0	3777	410	-68	3845	70	0	νOH
3747	145	-70	3818	70	1	3817	75	0	ν (N)OH
3540	26	11	3528	139	-1	3525	22	-4	νNH
3215	3	1	3213	4	-1	3216	3	2	νCH
3196	14	0	3194	14	-2	3197	12	1	νCH
3179	6	1	3178	10	0	3180	6	2	νCH
3154	13	1	3163	11	10	3155	11	2	νCH
2217	88	6	2233	95	22	2205	59	-6	νC≡O
1647	18	-1	1645	15	-3	1646	15	-2	νC=C
1637	27	-1	1638	38	0	1637	26	-1	νC=C
1540	71	0	1541	67	1	1540	70	0	δCH
1517	17	0	1516	24	-1	1517	20	0	δNH
1480	15	2	1476	20	-2	1478	16	0	δCH
1417	97	27	1391	80	1	1392	90	2	δNOH
1367	30	3	1370	63	6	1366	36	2	δOH
1336	12	0	1336	8	0	1336	9	0	δCH
1283	100	1	1284	92	2	1284	94	2	νC-O
1244	13	0	1254	37	10	1246	11	2	νC-N
1194	24	0	1203	74	9	1194	24	0	δCH
1183	62	0	1188	11	5	1184	60	1	δCH
1123	60	1	1128	36	6	1123	53	1	δCCC
1064	7	0	1064	7	0	1065	6	1	Ring def
1037	40	9	1028	46	0	1030	44	2	νN-O
975	0	3	971	0	-1	978	0	6	γCH
927	7	2	926	9	1	932	8	7	γCH
895	70	-9	906	90	2	905	98	1	γNH
851	1	2	851	0	2	852	0	1	γCH
829	27	-1	832	32	2	831	30	1	Ring def
760	68	-4	767	78	3	764	87	1	Skeleton def
749	81	2	748	71	1	750	65	3	γCH
720	64	-1	725	49	14	722	49	1	Skeleton def
610	5	0	610	4	0	610	5	0	Ring def
574	7	2	574	5	2	572	5	0	Skeleton def

536	17	4	536	7	4	533	8	1	δCCO, δCNO
523	4	1	524	1	2	522	3	0	Ring def
482	37	119	364	47	1	363	53	0	γ(N)OH
451	15	-4	454	5	-1	457	15	2	Skeleton def
346	5	2	348	19	4	346	12	2	δCCO
306	21	12	284	16	-10	299	32	5	Skeleton def
279	66	12	487	71	220	272	83	5	γOH
240	11	8	236	1	4	234	5	2	δCCN
188	1	13	175	4	0	178	2	3	Skeleton def
85	2	-9	100	1	6	100	1	6	Skeleton def
164	3		114	0		116	1		Intermolecular
141	2		90	3		61	0		Intermolecular
77	1		76	1		43	0		Intermolecular
27	0		20	0		27	0		Intermolecular
15	0		13	0		24	0		Intermolecular

Table S8 Anharmonic and harmonic wavenumbers (in cm^{-1}) calculated for the $C_6H_4(OH)NHOH$ molecule at the B3LYPD3/6-311++G(2d,2p) level of theory. The IR calculated intensities expressed in km mol^{-1}.

```
Fundamental Bands
 ------------------
   Mode(n)                  E(harm)   E(anharm)        I(harm)        I(anharm)
      1(1)                 3845.562   3629.031     70.03291390      57.40148829
      2(1)                 3817.146   3660.971     73.87447419      53.97756606
      3(1)                 3528.726   3352.747     22.30655332      16.01184062
      4(1)                 3214.445   3075.770      3.07157391       4.63788818
      5(1)                 3195.933   3060.680     12.97649148      11.70511696
      6(1)                 3178.506   3052.601      6.22885679       2.30830474
      7(1)                 3153.322   3014.221     12.55427677      17.16655432
      8(1)                 1646.828   1602.559     14.72724695       8.36648535
      9(1)                 1637.633   1598.341     26.78176042      16.97328312
     10(1)                 1539.822   1500.165     69.09927853      45.47272090
     11(1)                 1516.625   1478.616     19.24629956      10.07724612
     12(1)                 1476.676   1434.826     15.57987951       0.28275220
     13(1)                 1389.983   1343.017     92.11418637      62.57760047
     14(1)                 1363.841   1329.134     35.72949026      29.08938816
     15(1)                 1335.165   1305.569      8.48725017       5.39541908
     16(1)                 1281.597   1248.742    101.93112220      28.33835487
     17(1)                 1243.584   1227.789     10.31661494      41.37741896
     18(1)                 1193.645   1182.058     24.34945205      22.07972128
     19(1)                 1183.194   1147.092     61.49534593      24.21609417
     20(1)                 1122.132   1087.915     57.71043552      22.33904032
     21(1)                 1064.589   1043.242      6.39584836       4.31765498
     22(1)                 1028.411    993.380     45.01730983      22.87951683
     23(1)                  972.569    989.803      0.16502175       0.20518934
     24(1)                  925.281    932.621      9.24174783       3.26502114
     25(1)                  904.342    877.282     91.42895511      37.38238719
     26(1)                  848.605    855.491      0.21899413       0.50888204
```

Table S9 Energy of vertical excitations ΔE (nm) and corresponding oscillator strength (f) calculated using TD-DFT (B3LYP/6-311++G(2d,2p)) method.

ΔE (T1)	f	ΔE (T2)	f	ΔE (T3)	f	ΔE (S1)	f	ΔE (S2)	f	ΔE (S3)	f
381	0,0000	353	0.0000	314	0.0000	299	0.1282	262	0.0797	239	0.0181

Table S10 Anharmonic and harmonic wavenumbers (in cm^{-1}) calculated for the ketoketene $C_6H_4(=O)=C=O$ molecule at the B3LYP/6-311++G(2d,2p) level of theory. The IR calculated intensities expressed in km mol^{-1}.

Fundamental Bands

Mode(n)	E(harm)	E(anharm)	I(harm)	I(anharm)
1(1)	3206.709	3081.620	5.83142681	8.64617556
2(1)	3198.668	3067.235	3.89945653	1.22960971
3(1)	3185.989	3050.247	0.51408633	1.21189627
4(1)	3166.652	3030.426	9.59348811	11.94455114
5(1)	2202.384	2153.773	1225.31567440	958.25831626
6(1)	1683.909	1648.558	272.52330028	158.20964118
7(1)	1634.863	1607.001	125.06581792	66.63801302
8(1)	1547.781	1510.329	168.94892964	60.62955435
9(1)	1474.224	1443.174	11.13385439	10.49301439
10(1)	1418.771	1389.587	18.43913398	15.30126209
11(1)	1304.663	1277.866	59.80125123	36.00860124
12(1)	1262.472	1227.522	8.51999840	3.54310828
13(1)	1209.038	1188.406	13.97076650	8.08056204
14(1)	1171.478	1155.695	27.47922132	22.05849920
15(1)	1128.350	1107.185	7.68096720	4.44806698
16(1)	1009.869	1015.833	0.10006447	0.25792125
17(1)	997.641	982.706	3.80501001	3.00275482
18(1)	963.597	954.851	0.04072314	0.03864873
19(1)	869.154	856.020	4.72634146	4.60596900
20(1)	859.860	847.940	11.73136619	9.80400146
21(1)	757.091	751.768	31.15879732	12.83174301
22(1)	729.247	715.561	31.94725341	43.60905209
23(1)	700.370	689.648	17.66573945	16.10394382
24(1)	633.544	624.038	3.18583085	3.27107248
25(1)	578.973	569.868	12.52294870	12.06590013
26(1)	568.398	560.222	2.99706811	2.66652068
27(1)	491.723	489.047	8.23701758	7.50963924
28(1)	456.603	449.128	5.85256821	5.54001592
29(1)	383.902	381.410	0.26780715	0.45192955
30(1)	374.672	370.987	1.71875774	1.28179140
31(1)	192.576	188.478	1.84411006	1.79834317
32(1)	137.132	135.482	0.97632419	0.93706871
33(1)	87.742	86.048	1.22818034	1.22293744

Table S11 Selected structural parameters[a] calculated for the $C_6H_4(OH)NCO$-H_2O complexes at the B3LYPD3/6-311++G(2d,2p) level of theory.

Property	1a	1b	1c	1d
$r(C_4N_{13})$	1.404	1.405	1.406	1.406
$r(N_{13}C_{14})$	1.201	1.206	1.199	1.204
$r(C_{14}O_{15})$	1.171	1.167	1.173	1.169
$r(C_5O_{11})$	1.350	1.368	1.361	1.361
$r(O_{11}H_{12})$	0.975	0.966	0.965	0.965
$r(O_{18}H_{16})$	0.961	0.966	0.963	0.961
$r(O_{18}H_{17})$	0.962	0.960	0.960	0.961
$r(O_{11}H_{12}\cdots O_{18})$	1.867	-	-	-
$r(O_{18}H_{16}\cdots N_{13})$, $r(O_{18}H_{16}\cdots O_{11})$	2.802	2.006	-	-
$r(O_{18}H_{16}\cdots O_{15})$	3.567	-	2.276	-
$r(C_6H_{10}\cdots O_{18})$, $r(C_3H_9\cdots O_{18})$	-	2.527	2.399	2.596
$r(O_{11}H_{12}\cdots N_{13})$	2.377	2.200	2.226	2.216
$\phi(C_5O_{11}H_{12})$	113.2	108.9	108.7	108.6
$\phi(C_5C_4N_{13})$	117.9	116.5	116.6	116.6
$\phi(C_4N_{13}C_{14})$	139.0	137.1	139.7	137.9
$\phi(N_{13}C_{14}O_{15})$	173.6	174.3	174.1	174.3
$\phi(O_{11}H_{12}\cdots O_{18})$	161.0	-	-	-
$\phi(O_{18}H_{16}\cdots N_{13})$, $\phi(O_{18}H_{16}\cdots O_{11})$	97.9	159.1	-	-
$\phi(O_{18}H_{16}\cdots O_{15})$,	129.7	-	132.0	-
$\phi(C_6H_{10}\cdots O_{18})$, $\phi(C_3H_9\cdots O_{18})$		136.4	162.6	122.2
$\theta(O_{11}H_{12}\cdots O_{18}H_{16})$	140.1	171.8	-	-
$\theta(O_{18}H_{16}\cdots N_{13}C_{14})$	144.9	-	-173.3	-
$\theta(C_6H_{10}\cdots O_{18}H_{16})$, $\theta(C_3H_9\cdots O_{18}H_{16})$	-	-6.5	-8.5	116.8

[a] the interacting atoms refer to the structure 1a presented below;
the bond distances are given in Å, the angles in degrees.

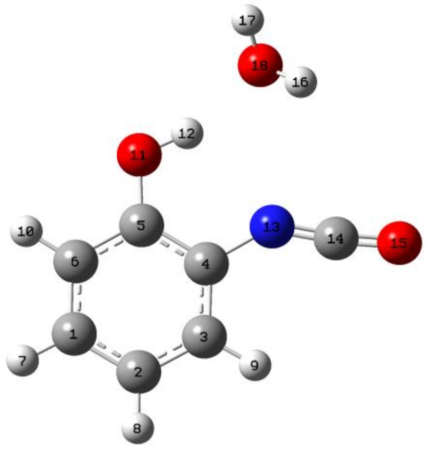

Table S12 Selected structural parameters[a] calculated for the $C_6H_4(OH)C(O)N$-H_2O complexes at the B3LYPD3/6-311++G(2d,2p) level of theory.

Property	2a	2b	2c
$r(C_4C_{13})$	1.438	1.438	1.437
$r(N_{13}C_{14})$	1.261	1.269	1.264
$r(C_{13}O_{12})$	1.329	1.315	1.321
$r(O_{12}N_{14})$	1.742	1.790	1.770
$r(C_5O_{11})$	1.342	1.352	1.360
$r(O_{11}H_{15})$	0.983	0.968	0.968
$r(O_{16}H_{17})$	0.961	0.962	0.960
$r(O_{16}H_{18})$	0.971	0.962	0.965
$r(O_{11}H_{15}\cdots O_{16})$	1.750	-	-
$r(O_{16}H_{18}\cdots N_{14})$, $r(O_{16}H_{18}\cdots O_{12})$, $r(O_{16}H_{18}\cdots O_{11})$	1.917	2.537	2.052
$r(C_6H_{10}\cdots O_{16})$, $r(C_3H_9\cdots O_{16})$	-	2.338	2.423
$r(O_{11}H_{15}\cdots O_{12})$	2.558	1.958	1.961
$\phi(C_5O_{11}H_{15})$	114.7	111.0	111.2
$\phi(C_5C_4C_{13})$	123.2	119.7	120.1
$\phi(C_4C_{13}N_{14})$	140.8	142.0	142.5
$\phi(C_4C_{13}O_{12})$	134.6	130.3	131.1
$\phi(C_{13}O_{12}N_{14})$	46.1	45.1	45.4
$\phi(O_{11}H_{15}\cdots O_{16})$	172.1	-	-
$\phi(O_{11}H_{15}\cdots O_{12})$	112.5	142.0	142.5
$\phi(O_{16}H_{18}\cdots N_{14})$, $\phi(O_{16}H_{18}\cdots O_{11})$		90.0	152.6
$\phi(O_{16}H_{18}\cdots O_{12})$,	136.5	-	-
$\phi(C_6H_{10}\cdots O_{16})$, $\phi(C_3H_9\cdots O_{16})$	-	154.6	139.2
$\theta(O_{11}H_{15}\cdots O_{16}H_{18})$	-177.8	-	160.3
$\theta(O_{16}H_{18}\cdots C_{13}N_{14})$	145.4	111.1	-
$\theta(C_6H_{10}\cdots O_{16}H_{18})$, $\theta(C_3H_9\cdots O_{16}H_{18})$	-	-57.6	-7.0

[a] the interacting atoms refer to the structure 1a presented below;
the bond distances are given in Å, the angles in degrees.

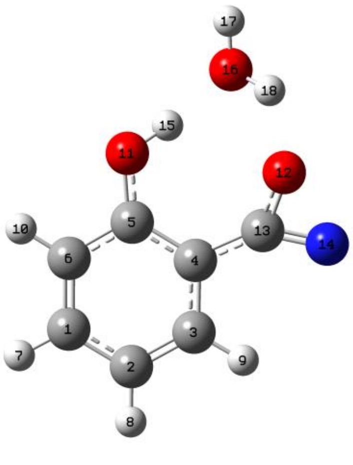

Table S13 Selected structural parameters[a] calculated for the $C_6H_4(OH)_2$-HNCO complexes at the B3LYPD3/6-311++G(2d,2p) level of theory.

Property	3a	3b	3c	3d
$r(N_{16}H_{15})$	1.010	1.015	1.010	1.012
$r(N_{16}C_{17})$	1.206	1.206	1.219	1.210
$r(C_{17}O_{15})$	1.171	1.169	1.159	1.166
$r(O_{11}H_{14})$	0.966	0.965	0.965	0.964
$r(O_{12}H_{13})$	0.965	0.961	0.969	0.962
$r(C_5O_{11})$	1.358	1.375	1.366	1.364
$r(C_4O_{12})$	1.381	1.377	1.373	1.390
$r(C_6H_{10})$	1.081	1.081	1.081	1.080
$r(O_{12}H_{13}\cdots O_{18})$, $r(O_{12}H_{13}\cdots N_{16})$	2.337	-	1.976	-
$r(N_{16}H_{15}\cdots C_1)$	2.695	-	-	-
$r(N_{16}H_{15}\cdots O_{11})$, $r(N_{16}H_{15}\cdots O_{12})$	-	1.911	-	2.021
$r(C_6H_{10}\cdots N_{16})$, $r(C_3H_9\cdots N_{16})$	-	3.026	2.893	-
$r(O_{11}H_{14}\cdots O_{12})$	2.164	2.159	2.154	2.214
$\phi(C_5O_{11}H_{14})$	107.8	108.9	108.2	109.4
$\phi(C_4O_{12}H_{13})$	109.5	110.5	110.9	110.2
$\phi(C_4C_5O_{11})$	120.6	120.2	120.5	121.6
$\phi(C_3C_4O_{12})$	123.4	124.5	124.4	123.5
$\phi(H_{15}N_{16}C_{17})$	123.4	127.3	122.6	124.5
$\phi(N_{16}C_{17}O_{18})$	173.4	173.4	173.3	173.1
$\phi(O_{12}H_{13}\cdots O_{18})$, $\phi(N_{16}H_{15}\cdots O_{12})$	132.1	-	-	164.5
$\phi(N_{16}H_{15}\cdots C_1)$, $\phi(N_{16}H_{15}\cdots O_{11})$	153.3	172.5	-	-
$\phi(O_{11}H_{14}\cdots O_{12})$, $\phi(O_{12}H_{13}\cdots N_{16})$	113.9	112.6	174.6	111.2
$\phi(C_6H_{10}\cdots N_{16})$, $\phi(C_3H_9\cdots N_{16})$	-	121.1	127.5	-
$\theta(C_1\cdots H_{15}N_{16}C_{17})$, $\theta(C_3H_9\cdots N_{16}H_{15})$,	7.1	-	98.5	-
$\theta(O_{12}H_{13}\cdots O_{18}C_{17})$, $\theta(N_{16}H_{15}\cdots O_{11}H_{14})$	21.4	157.2	-	107.0
$\theta(N_{16}H_{15}\cdots O_{12}H_{13})$				147.2
$\theta(O_{11}H_{14}\cdots O_{12}H_{13})$, $\theta(O_{12}H_{13}\cdots N_{16}H_{15})$,	-109.5	174.6	31.5	-132.7
$\theta(C_4O_{12}\cdots O_{18}C_{17})$, $\theta(C_6H_{10}\cdots H_{15}N_{16})$	-19.5	150.7	-	-

[a] the interacting atoms refer to the structure 1a presented below; the bond distances are given in Å, the angles in degrees.

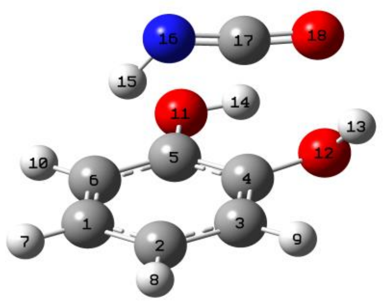

Table S14 Selected structural parameters[a] calculated for the $C_6H_4(OH)NHOH$-CO complexes at the B3LYPD3/6-311++G(2d,2p) level of theory.

Property	4a	4b	4c
$r(C_{17}O_{18})$	1.125	1.123	1.126
$r(C_5O_{11})$	1.377	1.373	1.373
$r(O_{11}H_{12})$	0.961	0.964	0.961
$r(C_4N_{13})$	1.409	1.411	1.410
$r(N_{13}H_{14})$	1.012	1.013	1.013
$r(N_{13}O_{15})$	1.423	1.431	1.430
$r(O_{15}H_{16})$	0.966	0.962	0.962
$r(O_{15}H_{16}\cdots C_{17})$, $r(O_{11}H_{12}\cdots C_{17})$, $r(C_{17}\cdots N_{13}H_{14})$	2.322	2.257	3.766
$r(C_3H_9\cdots O_{15})$	2.482	2.495	2.480
$r(N_{13}H_{14}\cdots O_{11})$	2.317	2.294	2.306
$\phi(C_5O_{11}H_{12})$	109.8	110.4	109.8
$\phi(C_4C_5O_{11})$	116.2	116.3	116.2
$\phi(C_4N_{13}H_{14})$	112.1	111.1	111.1
$\phi(O_{15}N_{13}H_{14})$	107.1	106.5	106.6
$\phi(C_4N_{13}O_{15})$	113.1	112.3	112.2
$\phi(N_{13}O_{15}H_{16})$	102.7	103.2	103.2
$\phi(O_{15}H_{16}\cdots C_{17})$, $\phi(O_{11}H_{12}\cdots C_{17})$	134.6	170.6	-
$\phi(N_{13}H_{14}\cdots O_{11})$	99.5	100.9	100.0
$\phi(C_3H_9\cdots O_{15})$, $\phi(C_{17}\cdots N_{13}H_{14})$	95.5	94.9	95.2
$\theta(H_{14}N_{13}O_{15}H_{16})$	121.1	116.5	116.1
$\theta(C_3C_4N_{13}H_{15})$	29.6	151.3	149.3
$\theta(C_4N_{13}O_{15}H_{16})$	-114.8	-121.7	-122.0
$\theta(N_{13}O_{15}H_{16}\cdots C_{17})$, $\theta(C_5O_{11}H_{12}\cdots C_{17})$	16.5	-173.0	60.8
$\theta(O_{15}H_{16}\cdots C_{17}O_{18})$, $\theta(O_{11}H_{12}\cdots C_{17}O_{18})$	-6.0	3.2	127.1
$\theta(N_{13}H_{14}\cdots O_{11}H_{12})$	-163.0	-162.2	-157.9
$\theta(C_3H_9\cdots O_{15}H_{16})$	124.7	127.4	126.2

[a] the interacting atoms refer to the structure 1a presented below; the bond distances are given in Å, the angles in degrees.

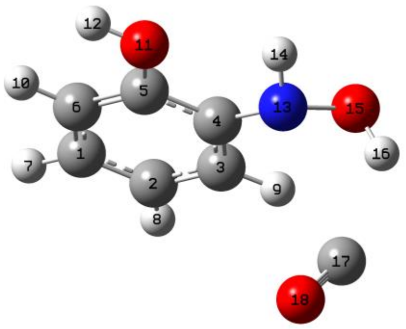

Supplement: Supplementary file 1 [file molecules-29-00862-s001.zip › molecules-2801801-supplementary.pdf]
